# Tear function in patients with diabetes mellitus: A systematic review and meta-analysis

**DOI:** 10.3389/fendo.2022.1036002

**Published:** 2022-10-21

**Authors:** Yu-Kai Kuo, Shih-Chieh Shao, Erh-Tsan Lin, Li-Yen Pan, Ling Yeung, Chi-Chin Sun

**Affiliations:** ^1^ Department of Ophthalmology, Keelung Chang Gung Memorial Hospital, Keelung, Taiwan; ^2^ School of Medicine, College of Medicine, Chang Gung University, Taoyuan, Taiwan; ^3^ Department of Pharmacy, Keelung Chang Gung Memorial Hospital, Keelung, Taiwan; ^4^ Department of Ophthalmology, Linkou Chang Gung Memorial Hospital, Taoyuan, Taiwan

**Keywords:** tear function, diabetes mellitus, dry eye, keratoconjunctivitis sicca, glycemic control

## Abstract

**Purpose:**

To examine tear function in patients with diabetes mellitus (DM).

**Design:**

Systematic review and meta-analysis.

**Method:**

We searched Embase and PubMed from database inception to March 16, 2022. We included observational studies that compared tear function between patients with and without DM. Tear function was measured using invasive tear breakup time (ITBUT) and Schirmer’s 1 test. Pooled results are presented as standard mean difference (SMD) with 95% confidence interval (CI) based on random-effects models.

**Results:**

We included 59 studies (7,234 eyes) comparing the tear function between patients with and without DM. This meta-analysis indicated that patients with DM had worse tear function than those without DM (ITBUT: SMD: −0.98, 95% CI: −1.27 to −0.69; Schirmer’s 1 test: SMD: −0.45, 95% CI: −0.64 to −0.26), and the results remained consistent in patients with different types of DM (e.g., type 1 DM and type 2 DM) and from different ethnic backgrounds (e.g., Asian vs. non-Asian). Patients with DM under poor glycemic control had worse tear function than those of the non-DM group (ITBUT: SMD: −1.26, 95% CI: −1.86 to −0.66; Schirmer’s 1 test: SMD: −0.25, 95% CI: −0.48 to −0.02), whereas there were no significant differences in tear function between patients with DM under optimal glycemic control and non-DM groups.

**Conclusions:**

We found that patients with type 1 or type 2 DM had significantly reduced tear function. The level of tear function could be determined by glycemic control, and therefore, our findings suggest that glycemic control in patients with DM is critical for maintaining tear function.

**Systematic Review Registration:**

https://www.crd.york.ac.uk/prospero, identifier CRD42021250498.

## Introduction

Diabetes mellitus (DM), a leading public health issue, affects more than 240 million people worldwide, and this number is expected to reach 370 million by 2030 (1). In addition to vascular complications, ocular complications of DM, such as dry eye disease (DED), diabetic retinopathy, glaucoma, and cataracts, negatively affect quality of life and may impose a huge economic burden (2). Among these ocular complications, DED occurs most frequently in patients with DM (3). For example, Seifart et al. reported that 52.8% of patients with DM suffered from DED compared with 9.3% in healthy controls (4).

Patients with DED often complain of a burning sensation, photopsia, foreign body sensation, soreness, itchiness, redness, and blurred vision. The corneal complications of DED include superficial punctate keratitis, neurotrophic keratopathy, and epithelial defects. In fact, both DM and DED are risk factors for corneal infection, scarring, perforation, and irreversible tissue damage (1). DM increases the risk of developing diabetic keratopathy, which presents as dry eye or recurrent erosions in the early or mild stage and neurotrophic ulcers with secondary infection in the advanced stage (5). In patients with DM, decreased lacrimal tear production results from being neurotrophic with loss of corneal sensation because of injury to the corneal receptors, which may further develop into a dry eye vicious cycle (6, 7).

A previous systematic review and meta-analysis by Lv et al. indicated that tear function is worse in patients with DM than in individuals without the disease, but we recommend that more detailed subgroup analyses should be considered to deal with the impacts of the clinical heterogeneity within the included studies, especially as regard different types of DM (8). For example, Kan et al. recently found no negative effects on tear function in patients with gestational DM (GDM) (9). This implies that different types of DM may cause varying pathophysiologies of DM-related DED. Furthermore, previous studies have reported that the corneal conditions in patients with DM may be determined by glycemic control, age, and ethnicity (10–12), but there is insufficient evidence to explore these factors in patients with DM-related DED.

In this study, we aimed to systematically examine the evidence on tear function in patients with DM; specifically, we evaluated tear function in these patients by conducting different subgroup analyses, including type of DM, age, ethnicity, and glycemic control status.

## Methods

This systematic review and meta-analysis followed the Preferred Reporting Items for Systematic Reviews and Meta-Analyses guidelines (**Supplemental Table S2**) (13). The study protocol has been registered on PROSPERO (CRD42021250498) (14).

### Search strategy and study selection

We searched Embase and PubMed for relevant records from the inception of these databases to March 16, 2022. The search strategy is presented in **Supplemental Table S3**. We also examined reference lists from previously published material and included studies from the lists to obtain further eligible studies. After potential records were identified from the abovementioned databases, two investigators (YKK and ETL) independently screened the study titles and abstracts. The same investigators selected studies by reviewing the full text based on our inclusion and exclusion criteria. Any disagreement about the study selection was resolved through full discussions with the third investigator (CCS).

### Eligibility criteria for study selection

Inclusion criteria for studies were as follows: (a) study groups included participants with DM, including type 1 DM, type 2 DM, GDM, and unclassified DM, and the control groups included participants without DM; (b) study outcome assessments used common tests to assess DED severity (15); and (c) study designs were cohort, case–control, or cross-sectional. We excluded studies in which (a) study or control groups focused on non-human participants; (b) study participants had Graves’ disease, connective tissue disorders, chronic kidney disease, or other autoimmune diseases (as autoimmune diseases disturb lacrimal secretion and dialysis alters tear quality) (16, 17); (c) study participants had a medical history of corneal disease, glaucoma, contact lens wearing, current use of ocular medication, or previous intraocular surgery (as structural damage to the cornea and eye drops interrupt tear secretion); (d) the literature was gray (e.g., conference abstracts) without detailed information on participants’ baseline characteristics, risk of bias evaluation, or results extraction; (e) data reports were duplicated (from the same source population); and (f) the language of publication was not English.

### Study outcomes

We included two tests for DED severity as study outcomes (18). First, Schirmer’s test is typically used to detect the amount of secretion of the aqueous layer of the tear film, and Schirmer’s 1 test measures total tear secretion function without topical anesthesia. Second, tear breakup time is used to determine the stability of the tear film, whereas invasive tear breakup time (ITBUT) is performed using strips soaked in fluorescein.

To evaluate differences in tear function within different subgroups of DM types, age, ethnicity, and DM control status, we further compared (1) tear function in the pertinent DM group with type 1 DM versus type 2 DM versus GDM versus unclassified DM; (2) mean participants’ age as those <65 versus >65 years old; (3) participants’ ethnicity as Asian versus non-Asian; and (4) mean glycosylated hemoglobin (HbA1C) levels as <7% versus >7%.

### Data extraction and quality assessment

Two investigators (YKK and ETL) independently extracted data regarding the study region, inclusion period, trial design, subgroups, sample size, mean age, sex ratio (male/female), DM duration, and HbA1C levels from the included studies. The outcome data for the meta-analyses included Schirmer’s 1 test and ITBUT. Because the outcomes of study interest were continuous data, we first extracted the mean and standard deviation (SD) from the included studies for the meta-analyses. If the included studies only reported the standard error (SE) or interquartile range (IQR), we calculated the SD using the formula SE = SD/√N and IQR/1.35, respectively (19, 20). If the included studies only reported the maximum and minimum values, we calculated the SD using the formula reported by Hozo et al. (21). If the studies only presented the subgroup data, we pooled them together into one group for the final meta-analysis.

Two investigators (YKK and ETL) independently assessed the study quality using an adapted form of the Newcastle–Ottawa Quality Assessment Scale for observational studies (22). This scale includes three major domains (selection, comparability, and outcome), with a total of 10 points. We defined studies with 7–10, 5–6, and 0–4 points as good, moderate, and low study quality, respectively (22). Any disagreement about the study quality assessments was resolved through full discussion with the third investigator (CCS).

### Statistical analysis

We conducted quantitative syntheses using meta-analysis to present the mean difference with a 95% confidence interval (CI) based on the random-effects model. We used Review Manager 5.4 software provided by the Cochrane Collaboration Network for the meta-analysis (23). We calculated the standard mean difference (SMD) to adjust for various measurement units from different measurement tools used among the included studies. We calculated I^2^ values to measure statistical heterogeneity among the studies. Furthermore, we performed subgroup analyses to evaluate the differences in tear function in patients with different types of DM, age, ethnicity, and HbA1C levels. We considered absolute SMDs of <0.2, 0.2–0.5, and >0.8 as small, medium, and large differences in DED severity, respectively, between the DM and control groups (24). Results with two-sided p < 0.05 were considered to be statistically significant.

## Results

**Figure 1:**
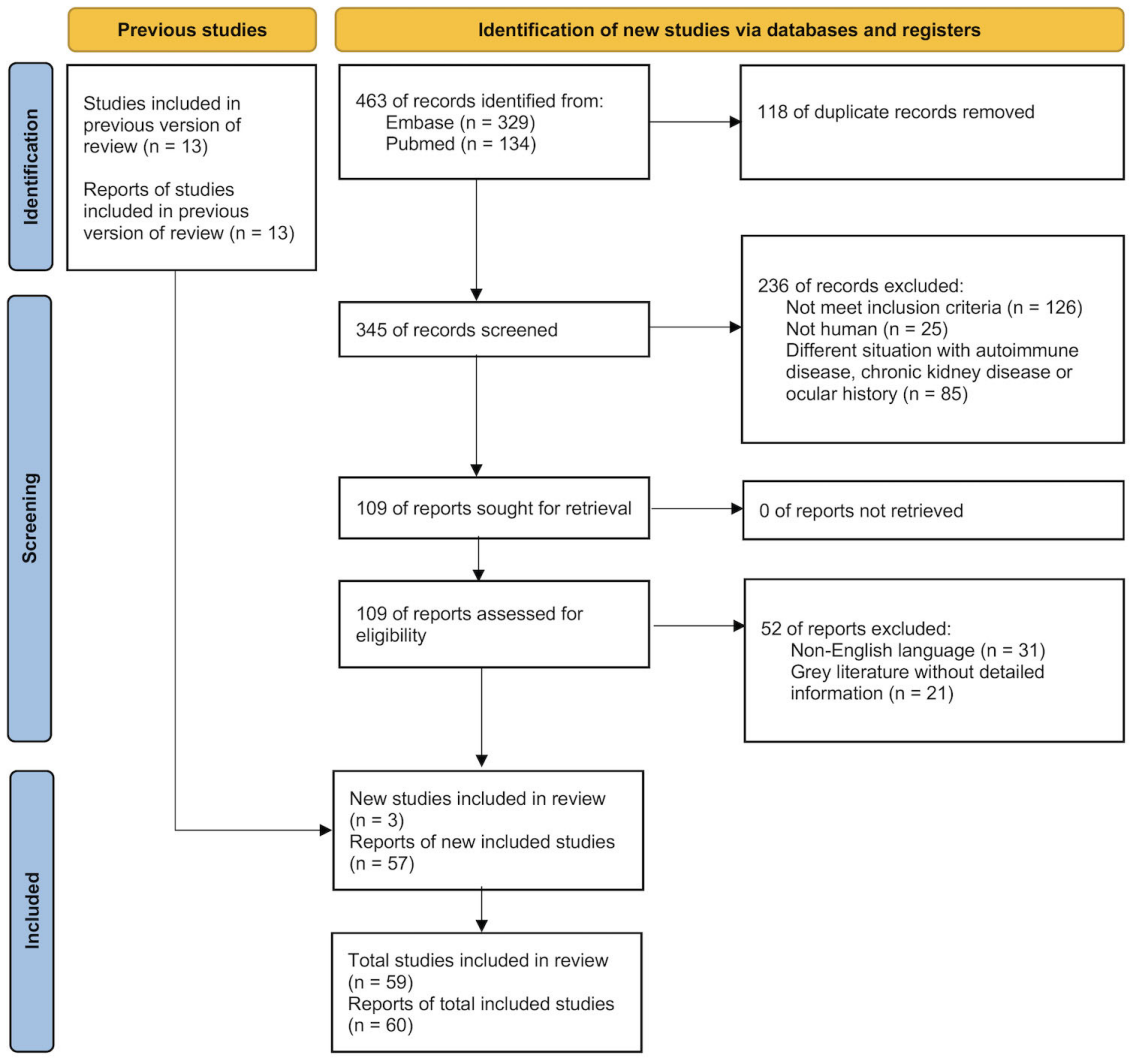
Flowchart of the systematic review with meta-analysis of the included studies.

We initially identified 466 records from Embase (n = 329), PubMed (n = 134), and three additional studies from the reference lists of previous literature. After applying our inclusion and exclusion criteria, we included 60 reports from 59 studies in this systematic review and meta-analysis (**Figure 1**). Specifically, the Zou et al. study presented two separate reports on adults (type 2 DM) and on children (either type 1 or 2 DM) (25). Hence, the meta-analysis included 60 reports from 59 studies.

### Characteristics and quality of included studies

The 59 studies contributed 7,234 eyes of participants with and without DM from China (20 studies, 2,780 eyes) (25–44), Turkey (8 studies, 891 eyes) (45–52), the United States (4 studies, 272 eyes) (9, 53–55), Japan (5 studies, 722 eyes) (56–60), Brazil (4 studies, 373 eyes) (61–64), the United Kingdom (2 studies, 117 eyes) (65, 66), India (3 studies, 569 eyes) (67–69), Korea (2 studies, 330 eyes) (70, 71), and other countries (11 studies, 1,180 eyes) (71–81). There were 1,210 (16.7%) eyes in the type 1 DM group, 4,345 (60.0%) in the type 2 DM group, 1,597 (22.1%) in the unclassified DM group, and 82 (1.1%) in the GDM group. Participants’ ages ranged from 10.1 ± 2.5 to 73.7 ± 5.7 years. The other study characteristics are listed in **Supplemental Table S1**.

### Methodological quality of included studies

Details of the risk of bias assessment are presented in **Supplemental Table S4**. All studies were assessed as having a low risk of bias, except for the domains of comparability. In general, the quality of the included studies was good.

### Main outcome

**Figure 2:**
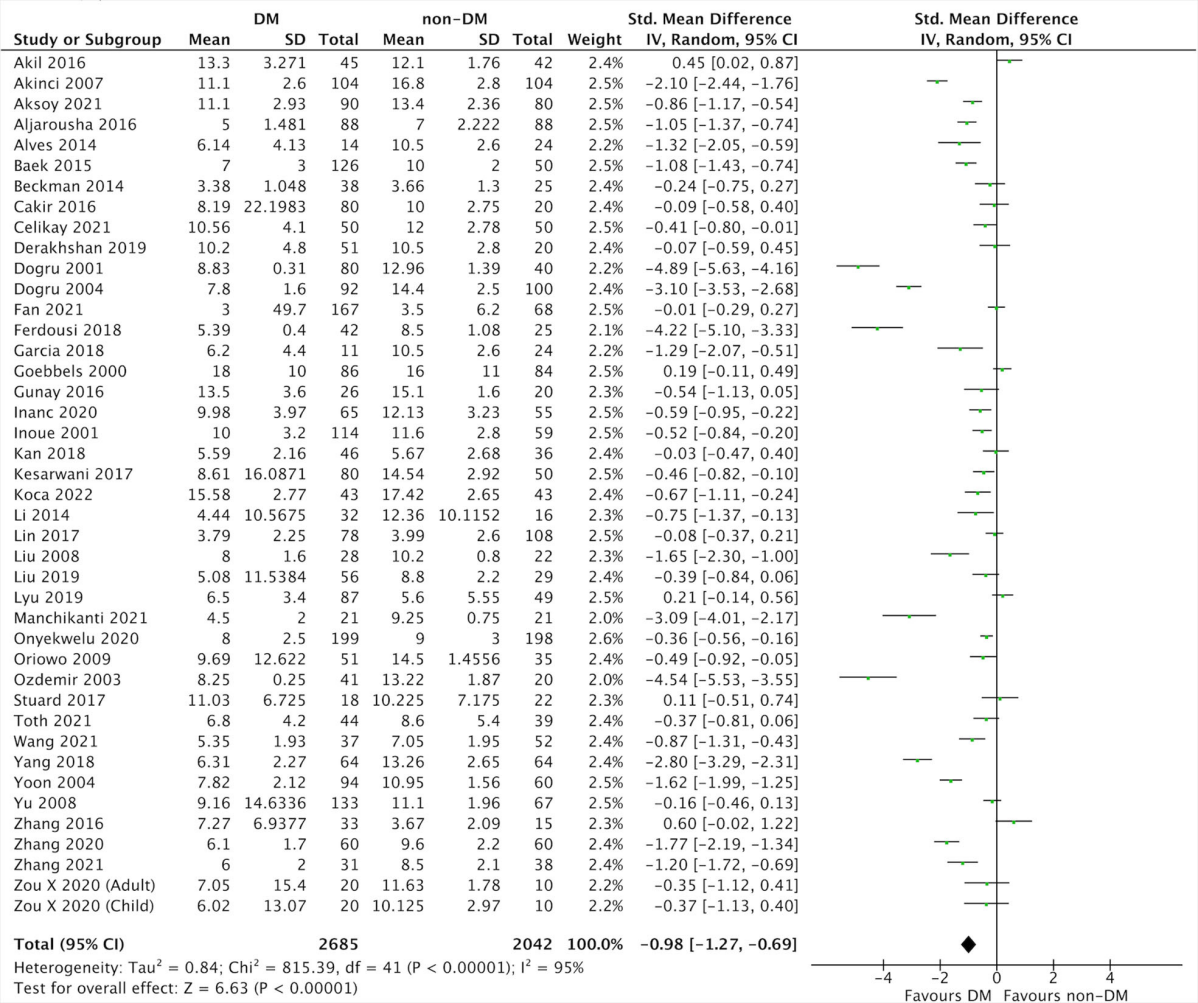
Comparison of the severity of dry eye disease (DED) between diabetes mellitus (DM) and non-DM based on invasive tear breakup time (ITBUT).

**Figure 3:**
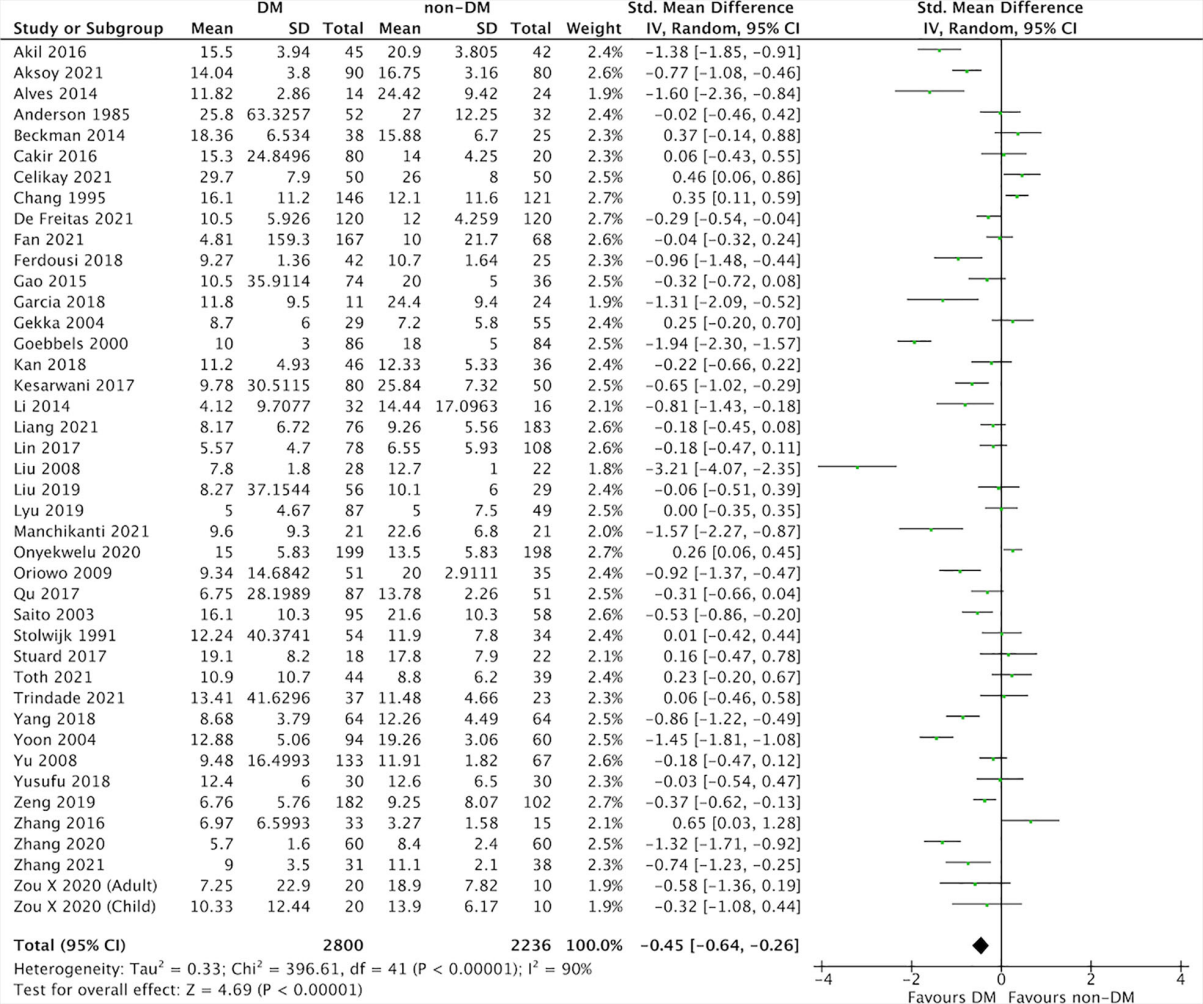
Comparison of the severity of dry eye disease (DED) between diabetes mellitus (DM) and non-DM based on Schirmer’s 1 test.

In this meta-analysis, 59 studies of DM evaluated severity of DED (**Figures 2** and **3**) (9, 25–82). Compared with the control group, we found that participants with DM had a lower ITBUT (41 studies, SMD: −0.98, 95% CI: −1.27 to −0.69, I^2^: 95%) and Schirmer’s 1 test result (41 studies, SMD: −0.45, 95% CI: −0.64 to −0.26, I^2^: 90%).

### Subgroup analysis stratified by different types of DM

This meta-analysis included 41 studies that evaluated ITBUT in relation to DM type (**Supplemental Figure S1A**) (9, 25, 28–32, 34, 35, 39–43, 45–57, 60, 62, 64, 65, 67–71, 73, 74, 79, 80, 82). Compared with the control group, we found lower ITBUTs in participants with type 1 DM (8 studies, SMD: −0.98, 95% CI: −1.70 to −0.26, I^2^: 96%), type 2 DM (21 studies, SMD: −1.26, 95% CI: −1.76 to −0.76, I^2^: 96%), and unclassified DM (12 studies, SMD: −0.59, 95% CI: −0.86 to −0.32, I^2^: 77%). No statistical differences in ITBUT were found in participants with GDM (one study, SMD: −0.03, 95% CI: −0.47 to 0.40, I^2^: not applicable).

There were 41 studies of DM where Schirmer’s 1 test results were evaluated (**Supplemental Figure S1B**) (9, 25, 26, 28–35, 37–39, 41–44, 47, 48, 51, 53–55, 58, 59, 61–65, 67–69, 71–75, 77, 82). Compared with the control group, we found lower Schirmer’s 1 test results in participants with type 1 DM (7 studies, SMD: −0.86, 95% CI: −1.39 to −0.33, I^2^: 91%) and type 2 DM (25 studies, SMD: −0.41, 95% CI: −0.63 to −0.18, I^2^: 88%). However, no statistical differences were found in participants with unclassified DM (nine studies, SMD: −0.24, 95% CI: −0.63 to 0.14, I^2^: 86%) or GDM (one study, SMD: −0.22, 95% CI: −0.66 to 0.22, I^2^: not applicable).

### Subgroup analysis stratified by age

**Supplemental Figure S2A** presents the 35 studies where ITBUT was evaluated in relation to age (9, 25, 28–32, 34, 39–43, 45–54, 56, 57, 60, 65, 67–71, 79, 80, 82). Compared with the control group, we found younger participants had a lower ITBUT (30 studies, SMD: −1.19, 95% CI: −1.55 to −0.83, I^2^: 96%), whereas no statistical differences in ITBUT were found among elderly participants (five studies, SMD: −0.27, 95% CI: −0.83 to 0.28, I^2^: 89%).

The 36 studies of DM where Schirmer’s 1 test results were evaluated in relation to age are shown in **Supplemental Figure S2B** (9, 25, 26, 28–34, 38, 39, 41–44, 47, 48, 51, 53, 54, 58, 59, 61, 63, 65, 67–69, 71–75, 77, 82). Compared with the control group, we found lower Schirmer’s 1 test results in younger participants (29 studies, SMD: −0.51, 95% CI: −0.76 to −0.26, I^2^: 92%) and elderly participants (7 studies, SMD: −0.23, 95% CI: −0.46 to −0.01, I^2^: 67%).

### Subgroup analysis stratified by ethnicity

There were 41 studies of DM that evaluated ITBUT together with ethnicity (**Supplemental Figure S3A**) (9, 25, 28–32, 34, 35, 39–43, 45, 47–57, 60, 62, 64, 65, 67–71, 73, 74, 79, 80, 82). Compared with the control group, we found a lower ITBUT in Asian (26 studies, SMD: −1.01, 95% CI: −1.35 to −0.66, I^2^: 95%) and non-Asian patients with DM (15 studies, SMD: −0.94, 95% CI: −1.49 to −0.39, I^2^: 95%).

With regard to Schirmer’s 1 test results, 41 studies included ethnicity (**Supplemental Figure S3B**) (9, 25, 26, 28–35, 37–39, 41–44, 47, 48, 51, 53–55, 58, 59, 61–65, 67–69, 71–75, 77, 82). Compared with the control group, we found lower Schirmer’s 1 test results in Asian (26 studies, SMD: −0.47, 95% CI: −0.69 to −0.26, I^2^: 89%) and non-Asian patients with DM (15 studies, SMD: −0.41, 95% CI: −0.80 to −0.02, I^2^: 91%).

### Subgroup analysis stratified by HbA1C levels

Twenty-one studies of DM investigated ITBUT in relation to HbA1C levels (**Supplemental Figure S4A**) (9, 30, 32, 34, 42, 45–51, 53, 54, 56, 60, 65, 69, 70, 80, 82). Compared with the control group, we found a lower ITBUT in participants with both poor control of DM (15 studies, SMD: −1.26, 95% CI: −1.86 to −0.66, I^2^: 97%) and good control of DM (6 studies, SMD: −0.47, 95% CI: −0.87 to −0.07, I^2^: 84%).

Twenty studies evaluated Schirmer’s 1 test results with HbA1C levels (**Supplemental Figure S4B**) (9, 30, 32–34, 38, 42, 47, 48, 51, 53, 54, 58, 59, 61, 63, 65, 69, 75, 82). Compared with the control group, we found lower Schirmer’s 1 test results in participants with poor control of DM (15 studies, SMD: −0.25, 95% CI: −0.48 to −0.02, I^2^: 83%), but no statistical differences were found in participants with good control of DM (5 studies, SMD: −0.25, 95% CI: −0.72 to 0.22, I^2^: 86%).

## Discussion

Based on the meta-analyses of ITBUT and Schirmer’s 1 tests, this study indicated that patients with DM presented with worse tear function than those without DM. More importantly, our findings could be the first summarized evidence on tear function within different DM subgroups. For example, unlike types 1 and 2 DM, we found patients with GDM had similar tear function to control groups. Moreover, patients with DM with good glycemic control had similar tear function to those without DM. However, tear function was similar in Asian and non-Asian patients with DM.

The influence of chronic hyperglycemia, such as in type 1 and type 2 DM, on DED has been elucidated by several mechanisms, including microvascular changes of the lacrimal gland, a reduced lipid layer in tear film composition, a high grade of conjunctival squamous metaplasia, an increased inflammatory process, and a low goblet cell density (83, 84). However, our subgroup analysis showed no significant difference between the tear function of patients with GDM and healthy pregnant women. A possible explanation could be the short duration of DM with a low degree of hyperglycemia in patients with GDM (9), so the clinical impacts from GDM on tear function may be relatively minor. Our finding may provide the fundamental evidence for further studies to confirm this proposed hypothesis.

Previous evidence regarding the role of ethnicity in tear function suggested that Asian populations were associated with higher risk of DED (12, 85, 86). However, in this systematic review and meta-analysis, we observed similar tear function in Asian and non-Asian patients with DM. Our findings may support the previous study from Butovich et al. indicating that minimal differences in meibogenesis and the process of lipid secretion from meibomian glands among different ethnicities were unlikely to differentially affect tear function between Asians and Caucasians (87).

Early studies reported age as a significant risk factor for decline of tear function, because it is associated with lacrimal gland atrophy with lymphocyte infiltration, eyelid laxity, and meibomian gland dysfunction (88–93). In this presented meta-analysis, we found elderly patients with DM may have better tear function than younger patients, contrary to previous reports. However, the impact of glycemic control on tear function in this subgroup analysis could not be ignored, because elderly patients usually have better glycemic control compared with younger patients (32, 42). Among five and seven included studies with elderly patients reporting ITBUT and Schirmer’s 1 test in our meta-analysis, respectively, only two studies reported the mean baseline HbA1C levels. We found both included studies had a mean HbA1C of less than 7%, whereas there were no differences in ITBUT (two studies, SMD: −0.48, 95% CI: −1.87 to 0.90, I^2^: 95%; **Supplemental Figure S5A**) and Schirmer’s 1 test results between this population and the controls (two studies, SMD: −0.35, 95% CI: −1.07 to 0.37, I^2^: 83%; **Supplemental Figure S5B**). Taking together all our results, we suggest that, under the optimal glycemic controls in elderly patients with DM could maintain the tear function as the control group. In addition, more studies on the tear function form elderly patients with inadequate glycemic controls should be determined.

Compared with previous systematic review with meta-analysis (8), this presented work included 46 more recent studies from China, Turkey, the United States, and other countries, which makes our findings more generalizable to clinical practice. However, some limitations should be noted before the interpretations of our study findings. First, we conducted various subgroup analyses (e.g., types of DM, age, ethnicity, and glycemic controls) with random-effects analyses to address the substantial clinical heterogeneity among the included studies. For example, some included studies were not based on well-matched designs to compare tear function between DM- and non-DM groups, so potential impacts from possible confounders could not be totally excluded. Second, result inconsistency among the studies were found, even after the subgroup stratifications with the random-effects analyses. Third, not every included study reported the mean with SD data for our meta-analysis; however, using different published approaches, we were able to convert SE, IQR, or maximum and minimum values. Finally, because this study mainly focused on type 1 or type 2 DM, our findings may not apply to prediabetic patients whose tear function may be substantially different from type 1 or type 2 DM patients (94). Regularly updated meta-analyses with future studies are required to replicate our findings.

In conclusion, this systematic review and meta-analysis found that patients with type 1 or type 2 DM had worse tear function compared with the non-DM groups. The level of tear function could be determined by glycemic control. Our findings suggest that glycemic control in patients with DM is critical for maintaining tear function.

## Data availability statement

The original contributions presented in the study are included in the article/**Supplementary Material**. Further inquiries can be directed to the corresponding author.

## Author contributions

Y-KK contributed to study planning, performed the systematic review search, and wrote the manuscript. S-CS performed study conception and meta-analysis and reviewed/edited the manuscript. E-TL contributed to study planning and implementation of supplemental analyses and reviewed/edited the manuscript. L-YP contributed to study planning and reviewed/edited the manuscript. LY contributed to study planning, wrote the methods, and reviewed/edited the manuscript. C-CS contributed to study conception and planning and reviewed/edited the manuscript. C-CS is the study guarantor. All authors contributed to the article and approved the submitted version.

## Funding

This research received the research grant MW#70986493 from Alcon Services AG, Taiwan Branch, Taiwan. The funder had no role in study design, data collection and analysis, decision to publish, or preparation of the manuscript.

## Conflict of interest

The authors declare that the research was conducted in the absence of any commercial or financial relationships that could be construed as a potential conflict of interest.

## Publisher’s note

All claims expressed in this article are solely those of the authors and do not necessarily represent those of their affiliated organizations, or those of the publisher, the editors and the reviewers. Any product that may be evaluated in this article, or claim that may be made by its manufacturer, is not guaranteed or endorsed by the publisher.

## References

[1] Sayın N Kara N Pekel G . Ocular complications of diabetes mellitus. World J Diabetes (2015) 6:92–108. doi: 10.4239/wjd.v6.i1.92 25685281 PMC4317321

[2] Vieira-Potter VJ Karamichos D Lee DJ . Ocular complications of diabetes and therapeutic approaches. BioMed Res Int (2016) 2016:3801570. doi: 10.1155/2016/3801570 27119078 PMC4826913

[3] Manaviat MR Rashidi M Afkhami-Ardekani M Shoja MR . Prevalence of dry eye syndrome and diabetic retinopathy in type 2 diabetic patients. BMC Ophthalmol (2008) 8:10–. doi: 10.1186/1471-2415-8-10 PMC2435518 18513455

[4] Seifart U Strempel I . [The dry eye and diabetes mellitus]. Ophthalmologe (1994) 91(2):235–9. 8012143

[5] Achtsidis V Eleftheriadou I Kozanidou E Voumvourakis KI Stamboulis E Theodosiadis PG . Dry eye syndrome in subjects with diabetes and association with neuropathy. Diabetes Care (2014) 37(10):e210–1. doi: 10.2337/dc14-0860 25249675

[6] Bikbova G Oshitari T Tawada A Yamamoto S . Corneal changes in diabetes mellitus. Curr Diabetes Rev (2012) 8(4):294–302. doi: 10.2174/157339912800840479 22587515

[7] Su YC Hung JH Chang KC Sun CC Huang YH Lee CN . Comparison of sodium-glucose cotransporter 2 inhibitors vs glucagonlike peptide-1 receptor agonists and incidence of dry eye disease in patients with type 2 diabetes in Taiwan. JAMA Netw Open (2022) 5(9):e2232584. doi: 10.1001/jamanetworkopen.2022.32584 36136333 PMC9500553

[8] Lv H Li A Zhang X Xu M Qiao Y Zhang J . Meta-analysis and review on the changes of tear function and corneal sensitivity in diabetic patients. Acta Ophthalmol (2014) 92(2):e96–e104. doi: 10.1111/aos.12063 23782539

[9] Kan S Acar U Kizilgul M Beyazyildiz E Cankaya AB Apaydin M . Tear film and ocular surface evaluation in gestational diabetes mellitus. Semin Ophthalmol (2018) 33(3):402–6. doi: 10.1080/08820538.2016.1250919 28005448

[10] Kim YJ Kim TG . The effects of type 2 diabetes mellitus on the corneal endothelium and central corneal thickness. Sci Rep (2021) 11(1):8324. doi: 10.1038/s41598-021-87896-3 33859349 PMC8050290

[11] Kaiserman I Kaiserman N Nakar S Vinker S . Dry eye in diabetic patients. Am J Ophthalmol (2005) 139(3):498–503. doi: 10.1016/j.ajo.2004.10.022 15767060

[12] Ward MF 2nd Le P Donaldson JC Van Buren E Lin FC Lefebvre C . Racial and ethnic differences in the association between diabetes mellitus and dry eye disease. Ophthal Epidemiol (2019) 26(5):295–300. doi: 10.1080/09286586.2019.1607882 31025588

[13] Page MJ McKenzie JE Bossuyt PM Boutron I Hoffmann TC Mulrow CD . The PRISMA 2020 statement: An updated guideline for reporting systematic reviews. Syst Rev (2021) 10(1):89. doi: 10.1186/s13643-021-01626-4 33781348 PMC8008539

[14] Booth A Clarke M Dooley G Ghersi D Moher D Petticrew M . PROSPERO: An international prospective register of systematic reviews. Syst Rev. (2012) 1:2. doi: 10.1186/2046-4053-1-2 22587842 PMC3348673

[15] Sullivan BD Whitmer D Nichols KK Tomlinson A Foulks GN Geerling G . An objective approach to dry eye disease severity. Invest Ophthalmol Visual Sci (2010) 51(12):6125–30. doi: 10.1167/iovs.10-5390 20631232

[16] Taskapili M Serefoglu Cabuk K Aydin R Atalay K Kirgiz A Sit D . The effects of hemodialysis on tear osmolarity. J Ophthalmol (2015) 2015:170361–. doi: 10.1155/2015/170361 PMC4657405 26640702

[17] Ziaragkali S Kotsalidou A Trakos N . Dry eye disease in routine rheumatology practice. Mediterr J Rheumatol (2018) 29(3):127–39. doi: 10.31138/mjr.29.3.127 PMC7046047 32185314

[18] Kuo YK Lin IC Chien LN Lin TY How YT Chen KH . Dry eye disease: A review of epidemiology in Taiwan, and its clinical treatment and merits. J Clin Med (2019) 8(8):1227. doi: 10.3390/jcm8081227 PMC6722537 31443274

[19] Fu R Vandermeer BW Shamliyan TA O’Neil ME Yazdi F Fox SH . Handling continuous outcomes in quantitative synthesis. (2013). 24006546

[20] Wan X Wang W Liu J Tong T . Estimating the sample mean and standard deviation from the sample size, median, range and/or interquartile range. BMC Med Res Methodol (2014) 14(1):135. doi: 10.1186/1471-2288-14-135 25524443 PMC4383202

[21] Hozo SP Djulbegovic B Hozo I . Estimating the mean and variance from the median, range, and the size of a sample. BMC Med Res Methodol (2005) 5:13. doi: 10.1186/1471-2288-5-13 15840177 PMC1097734

[22] Herzog R Álvarez-Pasquin MJ Díaz C Del Barrio JL Estrada JM Gil Á . Are healthcare workers’ intentions to vaccinate related to their knowledge, beliefs and attitudes? a systematic review. BMC Public Health (2013) 13(1):154. doi: 10.1186/1471-2458-13-154 23421987 PMC3602084

[23] Schwarzer G . Meta: An r package for meta-analysis. R News (2007) 7(3):40–5.

[24] Cohen J . A power primer. psychol Bull (1992) 112(1):155–9. doi: 10.1037/0033-2909.112.1.155 19565683

[25] Zou X Wang S Zhang P Lu L Zou H . Quantitative proteomics and weighted correlation network analysis of tear samples in adults and children with diabetes and dry eye. Trans Vision Sci Technol (2020) 9(13):1–15. doi: 10.1167/tvst.9.13.8 PMC7718812 33344052

[26] Gao Y Zhang Y Ru YS Wang XW Yang JZ Li CH . Ocular surface changes in type II diabetic patients with proliferative diabetic retinopathy. Int J Ophthalmol (2015) 8(2):358–64. doi: 10.3980/j.issn.2222-3959.2015.02.26 PMC4413579 25938056

[27] Han JX Wang H Liang HH Guo JX . Correlation of the retinopathy degree with the change of ocular surface and corneal nerve in patients with type 2 diabetes mellitus. Int J Ophthalmol (2021) 14(5):750–8. doi: 10.18240/ijo.2021.05.17 PMC8077005 34012892

[28] Li B Sheng M Xie L Liu F Yan G Wang W . Tear proteomic analysis of patients with type 2 diabetes and dry eye syndrome by two-dimensional nano-liquid chromatography coupled with tandem mass spectrometry. Invest Ophthalmol Visual Sci (2014) 55(1):177–86. doi: 10.1167/iovs.13-12080 24282230

[29] Lin X Xu B Zheng Y Coursey TG Zhao Y Li J . Meibomian gland dysfunction in type 2 diabetic patients. J Ophthalmol (2017) 2017:3047867. doi: 10.1155/2017/3047867 28593054 PMC5448054

[30] Liu R Ma B Gao Y Ma B Liu Y Qi H . Tear inflammatory cytokines analysis and clinical correlations in diabetes and nondiabetes with dry eye. Am J Ophthalmol (2019) 200:10–5. doi: 10.1016/j.ajo.2018.12.001 30552892

[31] Liu X Gu YS Xu YS . Changes of tear film and tear secretion after phacoemulsification in diabetic patients. J Zhejiang University: Sci B (2008) 9(4):324–8. doi: 10.1631/jzus.B0710359 PMC2276676 18381808

[32] Lyu Y Zeng X Li F Zhao S . The effect of the duration of diabetes on dry eye and corneal nerves. Contact Lens Anterior Eye (2019) 42(4):380–5. doi: 10.1016/j.clae.2019.02.011 31029535

[33] Qu JH Tian L Zhang XY Sun XG . Early central and peripheral corneal microstructural changes in type 2 diabetes mellitus patients identified using in vivo confocal microscopy: A case-control study. Med (United States) (2017) 96(38):e7960. doi: 10.1097/MD.0000000000007960 PMC5617699 28930832

[34] Yang M Zhao T Liu J Wang Z . Study on factors contributing to xerophthalmia among type-2 diabetes patients. Int J Clin Exp Med (2018) 11(4):4183–7.

[35] Yu L Chen X Qin G Xie H Lv P . Tear film function in type 2 diabetic patients with retinopathy. Ophthalmologica (2008) 222(4):284–91. doi: 10.1159/000140256 18560249

[36] Yu T Shi WY Song AP Gao Y Dang GF Ding G . Changes of meibomian glands in patients with type 2 diabetes mellitus. Int J Ophthalmol (2016) 9(12):1740–4. doi: 10.18240/ijo.2016.12.06 PMC5154985 28003972

[37] Yusufu M Liu X Zheng T Fan F Xu J Luo Y . Hydroxypropyl methylcellulose 2% for dry eye prevention during phacoemulsification in senile and diabetic patients. Int Ophthalmol (2018) 38(3):1261–73. doi: 10.1007/s10792-017-0590-7 28699061

[38] Zeng X Lv Y Gu Z Jia Z Zhang C Lu X . The effects of diabetic duration on lacrimal functional unit in patients with type II diabetes. J Ophthalmol (2019) 2019:8127515. doi: 10.1155/2019/8127515 30766731 PMC6350560

[39] Zhang M Xiang YH . Analysis of ocular surface dysfunction in patients with type 2 diabetes mellitus. Int Eye Sci (2020) 20(11):1853–7.

[40] Wang Y Li D Su W Dai Y . Clinical features, risk factors, and therapy of epithelial keratitis after cataract surgery. J Ophthalmol (2021) 2021:6636228. doi: 10.1155/2021/6636228 34035955 PMC8121559

[41] Zhang C Xi L Zhao S Wei R Huang Y Yang R . Interleukin-1β and tumour necrosis factor-α levels in conjunctiva of diabetic patients with symptomatic moderate dry eye: case-control study. BMJ Open (2016) 6(8):e010979. doi: 10.1136/bmjopen-2015-010979 PMC4985792 27489152

[42] Zhang K Zhang S Yu J Lu Y Zhu X . Changes of the tear film lipid layer thickness after cataract surgery in patients with diabetes mellitus. Acta Ophthalmol (2021) 99(2):e202–e8. doi: 10.1111/aos.14565 32749786

[43] Fan F Li X Li K Jia Z . To find out the relationship between levels of glycosylated hemoglobin with meibomian gland dysfunction in patients with type 2 diabetes. Ther Clin Risk Manage (2021) 17:797–807. doi: 10.2147/TCRM.S324423 PMC8355550 34393486

[44] Liang S Niu W Wang H Yang L . Alterations of ocular surface parameters in type 2 diabetic patients. Diabetes Metab Syndrome Obes: Targets Ther (2021) 14:3787–93. doi: 10.2147/DMSO.S323770 PMC8409513 34483673

[45] Akinci A Cetinkaya E Aycan Z . Dry eye syndrome in diabetic children. Eur J Ophthalmol (2007) 17(6):873–8. doi: 10.1177/112067210701700601 18050110

[46] Inanc M Kiziltoprak H Hekimoglu R Tekin K Ozalkak S Koc M . Alterations of tear film and ocular surface in children with type 1 diabetes mellitus. Ocular Immunol Inflamm (2020) 28(3):362–9. doi: 10.1080/09273948.2019.1571212 30806526

[47] Çakır BK Katırcıoğlu Y Ünlü N Duman S Üstün H . Ocular surface changes in patients treated with oral antidiabetic drugs or insulin. Eur J Ophthalmol (2016) 26(4):303–6. doi: 10.5301/ejo.5000710 26659019

[48] Celikay O Kosker M Çalışkan S Cakal E Pinarli FA Gurdal C . Ocular surface assessment in maturity-onset diabetes of the young. Int J Diabetes Develop Countries (2021) 41(1):136–40. doi: 10.1007/s13410-020-00843-2

[49] Ozdemir M Buyukbese MA Cetinkaya A Ozdemir G . Risk factors for ocular surface disorders in patients with diabetes mellitus. Diabetes Res Clin Pract (2003) 59(3):195–9. doi: 10.1016/S0168-8227(02)00244-9 12590016

[50] Gunay M Celik G Yildiz E Bardak H Koc N Kirmizibekmez H . Ocular surface characteristics in diabetic children. Curr Eye Res (2016) 41(12):1526–31. doi: 10.3109/02713683.2015.1136421 27159168

[51] Aksoy Aydemir G Aydemir E Asik A . Changes in tear meniscus analysis of children who have type 1 diabetes mellitus, with and without vitamin d deficiency. Cornea (2021) 41(11):1412–7. doi: 10.1097/ICO.0000000000002908 34812782

[52] Koca S Koca SB İnan S . Ocular surface alterations and changes of meibomian glands with meibography in type 1 diabetic children. Int Ophthalmol (2022) 42(5):1613–21. doi: 10.1007/s10792-021-02155-8 35088356

[53] Akil H Buluş AD Andiran N Alp MN . Ocular manifestations of type 1 diabetes mellitus in pediatric population. Indian J Ophthalmol (2016) 64(9):654–8. doi: 10.4103/0301-4738.194336 PMC5151155 27853013

[54] Stuard WL Titone R Robertson DM . Tear levels of insulin-like growth factor binding protein 3 correlate with subbasal nerve plexus changes in patients with type 2 diabetes mellitus. Invest Ophthalmol Visual Sci (2017) 58(14):6105–12. doi: 10.1167/iovs.17-22425 PMC5718599 29214310

[55] Beckman KA . Characterization of dry eye disease in diabetic patients versus nondiabetic patients. Cornea (2014) 33(8):851–4. doi: 10.1097/ICO.0000000000000163 24915011

[56] Dogru M Kaderli B Gelisken O Yücel A Avci R Goto E . Ocular surface changes with applanation contact lens and coupling fluid use after argon laser photocoagulation in noninsulin-dependent diabetes mellitus. Am J Ophthalmol (2004) 138(3):381–8. doi: 10.1016/j.ajo.2004.04.008 15364219

[57] Dogru M Katakami C Inoue M . Tear function and ocular surface changes in noninsulin-dependent diabetes mellitus. Ophthalmology (2001) 108(3):586–92. doi: 10.1016/S0161-6420(00)00599-6 11237914

[58] Saito J Enoki M Hara M Morishige N Chikama TI Nishida T . Correlation of corneal sensation, but not of basal or reflex tear secretion, with the stage of diabetic retinopathy. Cornea (2003) 22(1):15–8. doi: 10.1097/00003226-200301000-00004 12502941

[59] Gekka M Miyata K Nagai Y Nemoto S Sameshima T Tanabe T . Corneal epithelial barrier function in diabetic patients. Cornea (2004) 23(1):35–7. doi: 10.1097/00003226-200401000-00006 14701955

[60] Inoue K Kato S Ohara C Numaga J Amano S Oshika T . Ocular and systemic factors relevant to diabetic keratoepitheliopathy. Cornea (2001) 20(8):798–801. doi: 10.1097/00003226-200111000-00004 11685054

[61] Trindade M Castro de Vasconcelos J Ayub G Grupenmacher AT Gomes Huarachi DR Viturino M . Ocular manifestations and neuropathy in type 2 diabetes patients with charcot arthropathy. Front Endocrinol (2021) 12. doi: 10.3389/fendo.2021.585823 PMC8097086 33967949

[62] Alves M Reinach PS Paula JS Vellasco e Cruz AA Bachette L Faustino J . Comparison of diagnostic tests in distinct well-defined conditions related to dry eye disease. PloS One (2014) 9(5):e97921. doi: 10.1371/journal.pone.0097921 24848115 PMC4029783

[63] De Freitas GR Ferraz GAM Gehlen M Skare TL . Dry eyes in patients with diabetes mellitus. Prim Care Diabetes (2021) 15(1):184–6. doi: 10.1016/j.pcd.2020.01.011 32057723

[64] Garcia DM de Oliveira FR Módulo CM Faustino J Barbosa AP Alves M . Is sjögren’s syndrome dry eye similar to dry eye caused by other etiologies? discriminating different diseases by dry eye tests. PloS One (2018) 13(12):e0208420. doi: 10.1371/journal.pone.0208420 30507949 PMC6277094

[65] Ferdousi M Petropoulos IN Kalteniece A Azmi S Ponirakis G Efron N . No relation between the severity of corneal nerve, epithelial, and keratocyte cell morphology with measures of dry eye disease in type 1 diabetes. Invest Ophthalmol Vis Sci (2018) 59(13):5525–30. doi: 10.1167/iovs.18-25321 30480740

[66] Cousen P Cackett P Bennett H Swa K Dhillon B . Tear production and corneal sensitivity in diabetes. J Diabetes Complications (2007) 21(6):371–3. doi: 10.1016/j.jdiacomp.2006.05.008 17967709

[67] Kesarwani D Rizvi SWA Khan AA Amitava AK Vasenwala SM Siddiqui Z . Tear film and ocular surface dysfunction in diabetes mellitus in an Indian population. Indian J Ophthalmol (2017) 65(4):301–4. doi: 10.4103/ijo.IJO_939_15 PMC5452582 28513494

[68] Manchikanti V Kasturi N Rajappa M Gochhait D . Ocular surface disorder among adult patients with type II diabetes mellitus and its correlation with tear film markers: A pilot study. Taiwan J Ophthalmol (2021) 11(2):156–60. doi: 10.4103/tjo.tjo_56_20 PMC8259518 34295621

[69] Onyekwelu OM Aribaba OT Onyekwelu VI Idowu OO Salami MO Badmos KB . Correlation between clinical and cytological parameters of dry eye among diabetics in a Nigerian tertiary hospital. Int Ophthalmol (2020) 40(8):2055–64. doi: 10.1007/s10792-020-01382-9 32328917

[70] Baek J Doh SH Chung SK . Assessment of the tear meniscus using optical coherence tomography in patients with type 2 diabetes mellitus. Cornea (2015) 34(12):1534–40. doi: 10.1097/ICO.0000000000000651 26488630

[71] Yoon KC Im SK Seo MS . Changes of tear film and ocular surface in diabetes mellitus. Korean J Ophthalmol (2004) 18(2):168–74. doi: 10.3341/kjo.2004.18.2.168 15635831

[72] Andersen J Baun O Aamand HE . Tear secretion in juvenile diabetics with and without autonomic neuropathy. Acta Ophthalmol (1985) 63(1):93–6. doi: 10.1111/j.1755-3768.1985.tb05223.x 3993352

[73] Goebbels M . Tear secretion and tear film function in insulin dependent diabetics. Br J Ophthalmol (2000) 84(1):19–21. doi: 10.1136/bjo.84.1.19 10611093 PMC1723218

[74] Oriowo OM . Profile of central corneal thickness in diabetics with and without dry eye in a Saudi population. Optometry (2009) 80(8):442–6. doi: 10.1016/j.optm.2008.12.008 19635436

[75] Stolwijk TR van Best JA Lemkes HH de Keizer RJ Oosterhuis JA . Determination of basal tear turnover in insulin-dependent diabetes mellitus patients by fluorophotometry. Int Ophthalmol (1991) 15(6):377–82. doi: 10.1007/BF00137948 1778668

[76] Symeonidis C Papakonstantinou E Galli A Tsinopoulos I Mataftsi A Batzios S . Matrix metalloproteinase (MMP-2, -9) and tissue inhibitor (TIMP-1, -2) activity in tear samples of pediatric type 1 diabetic patients: MMPs in tear samples from type 1 diabetes. Graefe’s Arch Clin Exp Ophthalmol (2013) 251(3):741–9. doi: 10.1007/s00417-012-2221-3 23254483

[77] Chang SW Hsu HC Hu FR Chen MS . Corneal autofluorescence and epithelial barrier function in diabetic patients. Ophthal Res (1995) 27(2):74–9. doi: 10.1159/000267600 8538986

[78] Sandra Johanna GP Antonio LA Andrés GS . Correlation between type 2 diabetes, dry eye and meibomian glands dysfunction. J Optomet (2019) 12(4):256–62. doi: 10.1016/j.optom.2019.02.003 PMC6978584 31130447

[79] Aljarousha M Badarudin NE Che Azemin MZ . Comparison of dry eye parameters between diabetics and non-diabetics in district of kuantan, pahang. Malaysian J Med Sci (2016) 23(3):72–7. PMC4934721 27418872

[80] Derakhshan A Abrishami M Khajedaluee M Omidtabrizi A Moghaddam SG . Comparison between tear film osmolar cocentration and other tear film function parameters in patients with diabetes mellitus. Korean J Ophthalmol (2019) 33(4):326–32. doi: 10.3341/kjo.2013.0146 PMC6685821 31389208

[81] Eissa IM Khalil NM El-Gendy HA . A controlled study on the correlation between tear film volume and tear film stability in diabetic patients. J Ophthalmol (2016) 2016:5465272. doi: 10.1155/2016/5465272 27034823 PMC4789474

[82] Tóth N Silver DM Balla S Káplár M Csutak A . *In vivo* corneal confocal microscopy and optical coherence tomography on eyes of participants with type 2 diabetes mellitus and obese participants without diabetes. Graefes Arch Clin Exp Ophthalmol (2021) 259(11):3339–50. doi: 10.1007/s00417-021-05251-8 PMC8523500 34283292

[83] Najafi L Malek M Valojerdi AE Aghili R Khamseh ME Fallah AE . Dry eye and its correlation to diabetes microvascular complications in people with type 2 diabetes mellitus. J Diabetes Its Complications (2013) 27(5):459–62. doi: 10.1016/j.jdiacomp.2013.04.006 23726741

[84] Alves Mde C Carvalheira JB Módulo CM Rocha EM . Tear film and ocular surface changes in diabetes mellitus. Arq Bras Oftalmol (2008) 71(6 Suppl):96–103. doi: 10.1590/s0004-27492008000700018 19274419

[85] Craig JP Wang MT . Factors predisposing the Asian eye to dry eye disease. Invest Ophthalmol Visual Sci (2019) 60(9):2746–.

[86] Uchino M . What we know about the epidemiology of dry eye disease in Japan. Invest Ophthalmol Visual Sci (2018) 59(14):DES1–6. doi: 10.1167/iovs.17-23491 30481799

[87] Butovich IA Suzuki T Wojtowicz J Bhat N Yuksel S . Comprehensive profiling of Asian and Caucasian meibomian gland secretions reveals similar lipidomic signatures regardless of ethnicity. Sci Rep (2020) 10(1):14510. doi: 10.1038/s41598-020-71259-5 32883999 PMC7471331

[88] de Paiva CS . Effects of aging in dry eye. Int Ophthalmol Clin (2017) 57(2):47–64. doi: 10.1097/IIO.0000000000000170 PMC5347479 28282314

[89] Sharma A Hindman HB . Aging: A predisposition to dry eyes. J Ophthalmol (2014) 2014:781683. doi: 10.1155/2014/781683 25197560 PMC4150485

[90] Obata H Yamamoto S Horiuchi H Machinami R . Histopathologic study of human lacrimal gland. statistical analysis with special reference to aging. Ophthalmology (1995) 102(4):678–86. doi: 10.1016/S0161-6420(95)30971-2 7724184

[91] Bukhari AA Basheer NA Joharjy HI . Age, gender, and interracial variability of normal lacrimal gland volume using MRI. Ophthal Plast Reconstr Surg (2014) 30(5):388–91. doi: 10.1097/IOP.0000000000000117 24786180

[92] Chhadva P McClellan AL Alabiad C Feuer WJ Batawi H Galor A . Impact of eyelid laxity on symptoms and signs of dry eye disease. Cornea (2016) 35:531 5. doi: 10.1097/ICO.0000000000000786 26890664 PMC4779719

[93] Hykin PG Bron AJ . Age-related morphological changes in lid margin and meibomian gland anatomy. Cornea (1992) 11(4):334–42. doi: 10.1097/00003226-199207000-00012 1424655

[94] Richdale K Chao C Hamilton M . Eye care providers’ emerging roles in early detection of diabetes and management of diabetic changes to the ocular surface: A review. BMJ Open Diabetes Res Care (2020) 8(1):e001094. doi: 10.1136/bmjdrc-2019-001094 PMC7199150 32299899

