# Supplementary material for: Tear function in patients with diabetes mellitus: A systematic review and meta-analysis
Source: Front Endocrinol (Lausanne). 2022 Oct 21;13:1036002. doi: 10.3389/fendo.2022.1036002 (PMC9633841; doi:10.3389/fendo.2022.1036002)
Supplement: Supplementary file 1 [file DataSheet_1.docx]

Supplementary Material

# Supplementary Table 1. Characteristics of the included studies

| ***Supplementary Table 1.*** Characteristics of the Included Studies | | | | | | | | | |
| --- | --- | --- | --- | --- | --- | --- | --- | --- | --- |
| **Study** | **Region** | **Study period** | **Study design** | **Subgroups** | **Sample size** | **Age** | **Sex (male/female)** | **Duration of DM** | **Mean HbA1c level** |
|  |  |  |  |  | **[N (eyes)]** | **[years, mean ± SD]** | **[N]** | **[years, mean ± SD]** | **[%, mean ± SD]** |
| Akil et al. | California, | NR | Prospective | T1DM | 45 (45) | 13.2 ± 3.1 | 20/25 | 3.6 ± 3.1 | 9.7 ± 2.4 |
| (2016) | America |  | Case-control | CG | 42 (42) | 13.3 ± 2.6 | 21/21 |  |  |
| Akinci et al. | Ankara, | NR | Case-control | T1DM | 104 (104) | 12.3 ±5.8 | 57/47 | 6.1 ± 3.4 | 8.4 ± 3.4 |
| (2007) | Turkey |  |  | CG | 104 (104) | 12.7 ± 5.6 | 57/47 |  |  |
| Aksoy et al. | Adiyaman, | January/2021- | Prospective | T1DM | 90 (90) | 11.4 ± 2.8 | 31/59 | 6.1 ± 3.2 | 6.2 ± 1.1 |
| (2021) | Turkey | April/2021 | Cross-sectional | CG | 80 (80) | 12.1 ± 3.7 | 25/55 |  |  |
| Anderson et al. | Kolding, | NR | Cross-sectional | T1DM without AN | 26 (26) | 35 ± 6.75 | NR | 21 ± 6.5 | NR |
| (1985) | Denmark |  |  | T1DM with AN | 26 (26) | 39 ± 6.75 | NR | 20.5 ± 6.5 | NR |
|  |  |  |  | CG | 32 (32) | 30.5 ± 6.75 | NR |  |  |
| Ferdousi et al. | Manchester, | NR | Case-control | T1DM | 42 (42) | 49.2 ± 2.5 | 27/15 | 30.0 ± 2.6 | 8.5 ± 2.5 |
| (2018) | United Kingdom |  |  | CG | 25 (25) | 48.7 ± 2.8 | 11/14 |  | 5.3 ± 2.2 |
| Goebbels et al. | Herzogenrath, | NR | Case-control | T1DM | 86 (86) | 58 ± 8 | NR | NR | NR |
| (2000) | Germany |  |  | CG | 84 (84) | 60 ± 9 | NR |  |  |
| Inanc et al. | Van, | NR | Prospective | T1DM | 65 (65) | 14.2 ± 3.1 | 36/29 | 5.5 ± 3.3 | 6.3 ± 1.0 |
| (2020) | Turkey |  | Case-control | CG | 55 (55) | 13.9 ± 2.4 | 25/30 |  |  |
| Koca et al. | Afyonkarahisar, | NR | Prospective | T1DM | 43 (43) | NR | 18/25 | 6.8 ± 3.1 | NR |
| (2022) | Turkey |  | Case-control | CG | 43 (43) |  | 18/25 |  |  |
| Oriowo et al. | Riyadh, | NR | Retrospective | T1DM | 51 (51) | 53.9 ± 11.9 | 18/33 | NR | NR |
| (2009) | Saudi Arabia |  | Cross-sectional | CG | 35 (35) | 52.6 ± 10.7 | 17/18 |  |  |
| Stolwijk et al. | Leiden, | NR | Cross-sectional | T1DM without retinopathy | 25 (25) | 33.4 ± 12.1 | NR | 8.0 ± 6.6 | 7.7 ± 1.7 |
| (1991) | The Netherlands |  |  | T1DM with retinopathy | 29 (29) | 46.3 ± 10.4 | NR | 25.6 ± 7.1 | 8.2 ± 1.4 |
|  |  |  |  | CG | 34 (34) | 43.7 ± 16.5 | NR |  |  |
| Symeonidis et al. | Macedonia, | NR | Cross-sectional | T1DM | 27 (27) | 10.9 ± 3.0 | NR | 3.4 ± 2.5 | 8.1 ± 1.2 |
| (2013) | Greece |  |  | CG | 17 (17) | 10.1 ± 2.5 | NR |  |  |
| Baek et al. | Seoul, | NR | Cross-sectional | T2DM | 124 (124) | 63 ± 12 | 41/82 | 11 ± 8 | 7.9 ± 1.8 |
| (2015) | Korea |  |  | CG | 45 (45) | 59 ± 13 | 17/28 |  |  |
| Cakir et al. | Sakarya, | NR | Cross-sectional | T2DM with OAD | 20 (40) | 53.3 ± 6.8 | 8/12 | 7 | 7.6 ± 0.2 |
| (2016) | Turkey |  |  | T2DM with insulin | 20 (40) | 52.3 ± 7.0 | 6/14 | 6 | 7.7 ±0.2 |
|  |  |  |  | CG | 10 (20) | 51.6 ± 2.0 | 4/6 |  |  |
| Celikay et al. | Ankara, | January/2014- | Case-control | T2DM | 50 (50) | 25.6 ± 14.6 | 13/37 | 5.0 ± 5.5 | 5.5 ± 0.4 |
| (2021) | Turkey | January/2015 |  | CG | 50 (50) | 26.6 ± 12.7 | 14/36 |  | 4.9 ± 0.3 |
| Chang et al. | Taipei, | NR | Cross-sectional | T2DM | 146 (146) | 60.4 ± 6.8 | 77/69 | 11.7 ± 7.3 | NR |
| (1995) | Taiwan |  |  | CG | 121 (121) | 60.9 ± 9.5 | 61/60 |  |  |
| Cousen et al. | Edinburgh, | NR | Case-control | T2DM | 25 (25) | 61.8 ± 9.2 | 19/6 | NR | NR |
| (2007) | United Kingdom |  |  | CG | 25 (25) | 62.3 ± 10.8 | 19/6 |  |  |
| Dogru et al. | Chiba, | NR | Prospective | T2DM | 46 (92) | 54.9 ± 3.5 | 21/25 | 11.4 ± 4.7 | 9.78 ± 1.2 |
| (2004) | Japan |  | Case-control | CG | 50 (100) | 53.8 ± 4.2 | 26/24 |  |  |
| Dogru et al. | Kobe, | September/1998- | Prospective | T2DM | 50 (80) | 54.2 ± 5.2 | 24/26 | 13.6 ± 9.3 | NR |
| (2001) | Japan | February/1999 | Case-control | CG | 20 (40) | 56.4 ± 5.2 | 7/13 |  |  |
| Fan et al. | Hebei, | May/2018- | Cross-sectional | T2DM with HbA1C <7% | 60 (60) | 58.9 ± 10.0 | 36/24 | NR | NR |
| (2021) | China | December/2018 |  | T2DM with HbA1C > 7% | 107 (107) | 56.8 ± 10.0 | 62/45 | NR | NR |
|  |  |  |  | CG | 68 (68) | 58.4 ± 13.6 | 33/35 |  |  |
| Gao et al. | Shanxi, | December/2011- | Cross-sectional | T2DM | 58 (74) | 50 ± 8 | 27/31 | 9 ± 3 | NR |
| (2015) | China | May/2013 |  | CG | 36 (36) | 52 ± 8 | 21/15 |  |  |
| Han et al. | Jiangsu, | June/2019- | Cross-sectional | T2DM without DR | 33 (66) | 56.5 ± 7.5 | 16/17 | NR | NR |
| (2021) | China | August/2020 |  | T2DM with NPDR | 32 (64) | 58.6 ± 9.4 | 15/17 | NR | NR |
|  |  |  |  | T2DM with PDR | 34 (67) | 57.9 ± 8.2 | 17/17 | NR | NR |
|  |  |  |  | CG | 30 (60) | 56.4 ± 9.5 | 16/14 |  |  |
| Kesarwani et al. | Uttar Pradesh, | NR | Case-control | T2DM without DR | 29 (38) | 53.0 ± 5.6 | 14/15 | NR | NR |
| (2017) | India |  |  | T2DM with DR | 24 (42) | 52.5 ± 4.8 | 12/12 | NR | NR |
|  |  |  |  | CG | 30 (50) | 51.7 ± 4.8 | 15/15 |  |  |
| Li et al. | Shanghai, | NR | Case-control | T2DM without DE | 8 (16) | 59.6 ± 4.3 | 4/4 | 9.6 ± 2.5 | NR |
| (2014) | China |  |  | T2DM with DE | 8 (16) | 60.7 ± 4.3 | 4/4 | 12.4 ± 3.3 | NR |
|  |  |  |  | CG | 8 (16) | 60.6 ± 4.3 | 4/4 |  |  |
| Liang et al. | Jinan | December/2019- | Cross-sectional | T2DM | 38 (76) | 67.6 ± 9.1 | 16/22 | NR | NR |
| (2021) | China | November/2020 |  | CG | 92 (183) | 32.8 ±13.4 | 31/61 |  |  |
| Lin et al. | Zhejiang, | May/2015- | Prospective | T2DM | 39 (78) | 67.1 ± 1.5 | 16/23 | 9.1 ± 5.4 | NR |
| (2017) | China | December/2015 | Case-control | CG | 54 (108) | 67.2 ± 1.7 | 23/31 |  |  |
| Liu et al. | Beijing, | January/2018- | Case-control | T2DM without DE | 24 (24) | 63.5 ± 10.1 | 7/17 | 12.3 ± 6.8 | 8.0 ± 1.6 |
| (2019) | China | June/2018 |  | T2DM with DE | 32 (32) | 61.8 ± 9.8 | 14/18 | 11.3 ± 7.1 | 7.6 ± 1.5 |
|  |  |  |  | CG | 29 (29) | 62.4 ± 7.5 | 5/24 |  |  |
| Liu et al. | Hangzhou, | NR | Case-control | T2DM | 25 (28) | 64.3 | 16/9 | NR | NR |
| (2008) | China |  |  | CG | 22 (22) | 65.2 | 12/10 |  |  |
| Lyu et al. | Tianjin, | NR | Prospective | T2DM | 87 (87) | 65 ± 6 | 38/49 | 14 ± 8 | 6.9 ± 0.5 |
| (2019) | China |  | Case-control | CG | 49 (49) | 64 ± 5 | 17/32 |  |  |
| Manchikanti et al. | Puducherry, | July/2016- | Case-control | T2DM | 21 (21) | 54.6 ± 11.6 | 19/2 | NR | NR |
| (2021) | India | December/2017 |  | CG | 21 (21) | 51.3 ± 10.7 | 19/2 |  |  |
| Ozdemir et al. | Kahramanmaras, | September/2001- | Prospective | T2DM | 41 (41) | 53.2 ± 11.5 | 20/21 | 12.3 ± 6.8 | 8.2 ± 2.2 |
| (2003) | Turkey | March/2002 | Case-control | CG | 20 (20) | 55.5 ± 12.4 | 9/11 |  |  |
| Qu et al. | Beijing, | March/2015- | Prospective | T2DM without CFS | 48 (48) | 60.5 ± 8.4 | 14/34 | 13.4 ± 8.3 | 7.7 ± 1.1 |
| (2017) | China | November/2016 | Case-control | T2DM with CFS | 39 (39) | 63.8 ± 10.9 | 14/25 | 13.9 ± 5.2 | 7.8 ± 1.8 |
|  |  |  |  | CG | 51 (51) | 61.5 ± 10.2 | 18/33 |  |  |
| Saito et al. | Yamaguchi, | September/2000- | Cross-sectional | T2DM | 95 (95) | 65.4 ± 9.7 | 42/53 | 12.1 ± 8.0 | 7.2 ± 1.7 |
| (2003) | Japan | March/2001 |  | CG | 58 (58) | 64.4 ± 16.7 | 24/34 |  |  |
| Sandra et al. | Bogotà, | NR | Prospective | T2DM | 37 (37) | 59 ± 7.7 | 37/0 | 7.2 ± 5 | 6.8 ± 0.7 |
| (2019) | Colombia |  | Case-control | CG | 36 (36) | 58.5 ± 7.4 | 36/0 |  |  |
| Stuard et al. | Texas, | NR | Case-control | T2DM | 18 (18) | 58.8 ± 10.2 | 6/12 | NR | 7.7 ± 1.0 |
| (2017) | America |  |  | CG | 22 (22) | 53.3 ± 9.7 | 10/12 |  | 5.7 ± 0.4 |
| Toth et al. | Debrecen, | NR | Cross-sectional | T2DM | 44 (44) | 50 ± 7 | 26/18 | NR | 7.3 ± 1.1 |
| 2021 | Hungary |  |  | CG | 39 (39) | 53 ± 10 | 16/23 |  | 5.5 ± 0.3 |
| Trindade et al. | Sao Paulo, | January/2019- | Cross-sectional | T2DM without CA | 21 (21) | 60.6 ± 7.9 | 10/11 | 16.2 ± 8.7 | 8.7 ± 1.5 |
| (2021) | Brazil | September/2019 |  | T2DM with CA | 16 (16) | 57.1 ± 11.8 | 11/5 | 19 ± 10.1 | 8.5 ± 2.2 |
|  |  |  |  | CG | 23 (23) | 58.8 ± 9.6 | 9/14 |  |  |
| Yang et al. | Beijing, | October/2015- | Case-control | T2DM | 32 (64) | 53.2± 3.8 | 13/19 | NR | 9.6 ± 2.7 |
| (2018) | China | June/2017 |  | CG | 32 (64) | 54.7± 3.4 | 14/18 |  | 7.6 ± 2.5 |
| Yoon et al. | Gwang-Ju, | January/2003- | Cross-sectional | T2DM | 47 (94) | 55.7 ± 6.7 | 26/21 | NR | NR |
| (2004) | Korea | November/2003 |  | CG | 30 (60) | 53.5 ± 9.5 | 16/14 |  |  |
| Yu et al. | Chongqing, | NR | Case-control | T2DM with NPDR | 70 (70) | NR | 36/34 | NR | NR |
| (2008) | China |  |  | T2DM with PDR | 63 (63) | NR | 31/32 | NR | NR |
|  |  |  |  | CG | 67 (67) | NR | 32/35 |  |  |
| Yu et al. | Shandong, | October/2014- | Case-control | T2DM | 118 (118) | 59.7 ± 7.8 | 58/60 | NR | NR |
| (2016) | China | November/2015 |  | CG | 100 (100) | 60.3 ± 7.6 | 52/48 |  |  |
| Yusufu et al. | Shanghai, | December/2014- | Prospective | T2DM | 30 (30) | NR | NR | NR | NR |
| (2018) | China | May/2015 | Case-control | CG | 30 (30) |  |  |  |  |
| Zeng et al. | Tianjin, | July/2017- | Prospective | T2DM | 91 (182) | 65.4 ± 6.3 | 40/51 | 13.6 ± 8.3 | 7.0 ± 0.5 |
| (2019) | China | September/2017 | Case-control | CG | 51 (102) | 64.4 ± 5.7 | 18/33 |  |  |
| Zhang et al. | Beijing, | NR | Case-control | T2DM | 60 (60) | 63.6 ± 11.0 | 32/28 | NR | NR |
| (2020) | China |  |  | CG | 60 (60) | 63.4 ± 10.4 | 30/30 |  |  |
| Zou et al. | Shanghai, | August/2017 | Cross-sectional | T2DM without DE | 10 (10) | 57.7 ± 7.2 | 3/7 | 5.7 ± 3.0 | NR |
| (2020) (Adult) | China |  |  | T2DM with DE | 10 (10) | 58.8 ±4.3 | 4/6 | 12.4 ± 4.5 | NR |
|  |  |  |  | CG | 10 (10) | 58.0 ± 4.3 | 3/7 |  |  |
| Aljarousha et al. | Pahang, | October/2007- | Retrospective | Unclassified DM | 88 (88) | 55 ± 10.1 | 38/50 | NR | NR |
| (2016) | Malaysia | October/2013 | Case-control | CG | 88 (88) | 53 ± 7.3 | 38/50 |  |  |
| Alves et al. | São Paulo, | NR | Case-control | Unclassified DM | 14 (14) | NR | NR | NR | NR |
| (2014) | Brazil |  |  | CG | 24 (24) |  |  |  |  |
| Beckman et al. | Ohio, | NR | Cross-sectional | Unclassified DM | 38 (38) | NR | 16/22 | 11.1 ± 9.0 | NR |
| (2014) | America |  |  | CG | 25 (25) |  | 4/21 |  |  |
| De Freitas et al. | Paraná, | April/2018- | Case-control | Unclassified DM | 120 (120) | 59 ± 7.3 | 51/69 | 10 ± 9.6 | 8.1 ± 2.1 |
| (2021) | Brazil | March/2019 |  | CG | 120 (120) | 57 ± 15.4 | 54/66 |  |  |
| Derakhshan et al. | Mashhad, | September/2011- | Case-control | Unclassified DM | 51 (51) | 51.2 ± 12.5 | 16/35 | 10.9 ± 7.1 | 10.1 ± 1.9 |
| (2019) | Iran | 2012 |  | CG | 20 (20) | 48.5 ± 12.4 | 9/11 |  |  |
| Eissa et al. | Cairo. | NR | Prospective | Unclassified DM | 22 (22) | 57.3 ± 8.5 | 9/13 | 13.5 ± 7.5 | NR |
| (2016) | Egypt |  | Case-control | CG | 16 (16) | 57.1 ± 12.2 | 7/9 |  |  |
| Garcia et al. | São Paulo, | NR | Case-control | Unclassified DM | 11 (11) | NR | NR | NR | NR |
| (2018) | Brazil |  |  | CG | 24 (24) |  |  |  |  |
| Gekka et al. | Tokyo, | NR | Cross-sectional | Unclassified DM | 29 (29) | 56.7 ± 11.7 | NR | 11.3 ± 9.3 | 8.7 ± 2.0 |
| (2004) | Japan |  |  | CG | 55 (55) | 60.6 ± 11.7 |  |  |  |
| Gunay et al. | Istanbul, | December/2014- | Prospective | Unclassified DM | 26 (26) | 11.8 ± 3.1 | 14/12 | 2.4 ± 1.6 | 8.6 ± 1.4 |
| (2016) | Turkey | June/2015 | Case-control | CG | 20 (20) | 11.6 ± 2.7 | 12/8 |  |  |
| Inoue et al. | Tokyo, | September/1999- | Cross-sectional | Unclassified DM | 114 (114) | 63.8 ± 11.1 | 65/49 | 13.6 ± 9.4 | 7.8 ± 1.7 |
| (2001) | Japan | November/1999 |  | CG | 59 (59) | 63.0 ± 11.1 | 24/35 |  |  |
| Onyekwelu et al. | Tamil Nadu | September/2016- | Case-control | Unclassified DM | 199 | 58.5 ± 10.1 | 87/121 | 9.5±8.3 | 7.4±1.8 |
| (2020) | India | December/2016 |  | CG | 198 | 58.3 ± 10.5 |  |  |  |
| Wang et al. | Qingdao, | January/2018- | Retrospective | Unclassified DM | 37 (37) | 73.7 ± 5.7 | NR | NR | NR |
| (2021) | China | December/2019 | Cross-sectional | CG | 52 (52) | 66.8 ± 9.9 | NR |  |  |
| Zhang et al. | Tianjin, | NR | Case-control | Unclassified DM without DE | 14 (14) | 67.9 ± 7.4 | 6/8 | NR | NR |
| (2016) | China |  |  | Unclassified with DE | 19 (19) | 65.4 ± 9.0 | 9/10 | NR | NR |
|  |  |  |  | CG | 14 (15) | 68.9 ± 9.6 | 8/6 |  |  |
| Zhang et al. | Shanghai, | May/2019- | Prospective | Unclassified DM | 31 (31) | 65.2 ± 11.0 | 13/18 | 11.0 ± 7.3 | 6.6 ± 1.2 |
| (2021) | China | July/2019 | Case-control | CG | 38 (38) | 71.0 ± 10.7 | 17/21 |  | 5.7 ± 0.5 |
| Zou et al. | Shanghai, | January/2018- | Cross-sectional | Unclassified DM without DE | 10 (10) | 12.0 ± 3.3 | 4/6 | 4.1 ± 3.0 | NR |
| (2020) (Child) | China | February/2018 |  | Unclassified DM with DE | 10 (10) | 11.7 ± 2.8 | 4/6 | 3.6 ± 3.7 | NR |
|  |  |  |  | CG | 10 (10) | 11.2 ± 1.3 | 4/6 |  |  |
| Kan et al. | Minneapolis, | NR | Case-control | GDM | 46 (46) | 30.4 ± 4.8 | 0/46 | NR | 5.6 ± 0.5 |
| (2018) | America |  |  | CG | 36 (36) | 28.8 ± 4.4 | 0/36 |  | 4.9 ± 0.3 |

T1DM, type 1 diabetes mellitus (insulin-dependent diabetes mellitus); T2DM, type 2 diabetes mellitus (non-insulin-dependent diabetes mellitus); DM, diabetes mellitus; GDM, gestational diabetes mellitus; CG, control group; HbA1C, glycosylated hemoglobin; AN, autonomic neuropathy; OAD, oral antidiabetic drugs; DR, diabetic retinopathy; DE, dry eye; CFS, cornea fluorescein stain; NPDR, non-proliferative diabetic retinopathy; PDR, proliferative diabetic retinopathy.

# Supplementary Table 2. PRISMA 2020 Checklist.

| **Section and Topic** | **Item #** | **Checklist item** | **Location where item is reported** |
| --- | --- | --- | --- |
| **TITLE** | | |  |
| Title | 1 | Identify the report as a systematic review. | Page 1 |
| **ABSTRACT** | | |  |
| Abstract | 2 | See the PRISMA 2020 for Abstracts checklist. | Page 3 |
| **INTRODUCTION** | | |  |
| Rationale | 3 | Describe the rationale for the review in the context of existing knowledge. | Page 5 |
| Objectives | 4 | Provide an explicit statement of the objective(s) or question(s) the review addresses. | Page 5 |
| **METHODS** | | |  |
| Eligibility criteria | 5 | Specify the inclusion and exclusion criteria for the review and how studies were grouped for the syntheses. | Page 7 |
| Information sources | 6 | Specify all databases, registers, websites, organisations, reference lists and other sources searched or consulted to identify studies. Specify the date when each source was last searched or consulted. | Page 7 |
| Search strategy | 7 | Present the full search strategies for all databases, registers and websites, including any filters and limits used. | Supplementary Table 1. |
| Selection process | 8 | Specify the methods used to decide whether a study met the inclusion criteria of the review, including how many reviewers screened each record and each report retrieved, whether they worked independently, and if applicable, details of automation tools used in the process. | Page 8 |
| Data collection process | 9 | Specify the methods used to collect data from reports, including how many reviewers collected data from each report, whether they worked independently, any processes for obtaining or confirming data from study investigators, and if applicable, details of automation tools used in the process. | Page 8 |
| Data items | 10a | List and define all outcomes for which data were sought. Specify whether all results that were compatible with each outcome domain in each study were sought (e.g. for all measures, time points, analyses), and if not, the methods used to decide which results to collect. | Page 8 |
|  | 10b | List and define all other variables for which data were sought (e.g. participant and intervention characteristics, funding sources). Describe any assumptions made about any missing or unclear information. | NR |
| Study risk of bias assessment | 11 | Specify the methods used to assess risk of bias in the included studies, including details of the tool(s) used, how many reviewers assessed each study and whether they worked independently, and if applicable, details of automation tools used in the process. | Page 9 |
| Effect measures | 12 | Specify for each outcome the effect measure(s) (e.g. risk ratio, mean difference) used in the synthesis or presentation of results. | Page 9 |
| Synthesis methods | 13a | Describe the processes used to decide which studies were eligible for each synthesis (e.g. tabulating the study intervention characteristics and comparing against the planned groups for each synthesis (item #5)). | Page 7 |
|  | 13b | Describe any methods required to prepare the data for presentation or synthesis, such as handling of missing summary statistics, or data conversions. | Page 8 |
|  | 13c | Describe any methods used to tabulate or visually display results of individual studies and syntheses. | NR |
|  | 13d | Describe any methods used to synthesize results and provide a rationale for the choice(s). If meta-analysis was performed, describe the model(s), method(s) to identify the presence and extent of statistical heterogeneity, and software package(s) used. | NR |
|  | 13e | Describe any methods used to explore possible causes of heterogeneity among study results (e.g. subgroup analysis, meta-regression). | Page 8 |
|  | 13f | Describe any sensitivity analyses conducted to assess robustness of the synthesized results. | NR |
| Reporting bias assessment | 14 | Describe any methods used to assess risk of bias due to missing results in a synthesis (arising from reporting biases). | Page 9 |
| Certainty assessment | 15 | Describe any methods used to assess certainty (or confidence) in the body of evidence for an outcome. | NR |
| **RESULTS** | | |  |
| Study selection | 16a | Describe the results of the search and selection process, from the number of records identified in the search to the number of studies included in the review, ideally using a flow diagram. | Figure 1. |
|  | 16b | Cite studies that might appear to meet the inclusion criteria, but which were excluded, and explain why they were excluded. | NR |
| Study characteristics | 17 | Cite each included study and present its characteristics. | Table 1. |
| Risk of bias in studies | 18 | Present assessments of risk of bias for each included study. | Supplementary Table 2. |
| Results of individual studies | 19 | For all outcomes, present, for each study: (a) summary statistics for each group (where appropriate) and (b) an effect estimate and its precision (e.g. confidence/credible interval), ideally using structured tables or plots. | NR |
| Results of syntheses | 20a | For each synthesis, briefly summarise the characteristics and risk of bias among contributing studies. | Page 11 |
|  | 20b | Present results of all statistical syntheses conducted. If meta-analysis was done, present for each the summary estimate and its precision (e.g. confidence/credible interval) and measures of statistical heterogeneity. If comparing groups, describe the direction of the effect. | Page 11 |
|  | 20c | Present results of all investigations of possible causes of heterogeneity among study results. | NR |
|  | 20d | Present results of all sensitivity analyses conducted to assess the robustness of the synthesized results. | NR |
| Reporting biases | 21 | Present assessments of risk of bias due to missing results (arising from reporting biases) for each synthesis assessed. | NR |
| Certainty of evidence | 22 | Present assessments of certainty (or confidence) in the body of evidence for each outcome assessed. | NR |
| **DISCUSSION** | | |  |
| Discussion | 23a | Provide a general interpretation of the results in the context of other evidence. | Page 15 |
|  | 23b | Discuss any limitations of the evidence included in the review. | Page 16 |
|  | 23c | Discuss any limitations of the review processes used. | Page 16 |
|  | 23d | Discuss implications of the results for practice, policy, and future research. | Page 16 |
| **OTHER INFORMATION** | | |  |
| Registration and protocol | 24a | Provide registration information for the review, including register name and registration number, or state that the review was not registered. | Page 7 |
|  | 24b | Indicate where the review protocol can be accessed, or state that a protocol was not prepared. | Page 7 |
|  | 24c | Describe and explain any amendments to information provided at registration or in the protocol. | NR |
| Support | 25 | Describe sources of financial or non-financial support for the review, and the role of the funders or sponsors in the review. | Page 1 |
| Competing interests | 26 | Declare any competing interests of review authors. | Page 1 |
| Availability of data, code and other materials | 27 | Report which of the following are publicly available and where they can be found: template data collection forms; data extracted from included studies; data used for all analyses; analytic code; any other materials used in the review. | NR |

# Supplementary Table 3. Search strategy.

| PubMed | ('diabetes mellitus'/exp OR 'diabetes' OR 'diabetes mellitus' OR 'diabetic') AND ('schirmer test'/exp OR 'schirmer test' OR 'schirmer tear test' OR 'test, schirmer' OR 'tear break-up time'/exp OR 'but (tear break-up time)' OR 'tbut (tear break-up time)' OR 'tear break-up time' OR 'tear breakup time' OR 'tear film break-up time' OR 'tear film breakup time') |
| --- | --- |
| Embase | (("tear break-up time" OR "tear breakup time" OR 'tear film break-up time' OR "tear film breakup time") OR ("Schirmer test" or "Schirmer's test" or "Schirmer tear test")) AND ("diabetes mellitus"[MeSH Terms] OR ("diabetes"[All Fields] AND "mellitus"[All Fields]) OR "diabetes mellitus"[All Fields] OR "diabetes"[All Fields] OR "diabetic"[All Fields] OR "diabetics"[All Fields] OR "diabets"[All Fields]) |

# Supplementary Table 4. Study quality of literatures included in the meta-analysis.

| Study | Selection | | | | | Comparability | | Exposure/Outcome | | | Total Number of Stars |
| --- | --- | --- | --- | --- | --- | --- | --- | --- | --- | --- | --- |
| Akil et al, 2016 | ★ | ☆ | ★ | ★ | ★ | ★ | ☆ | ★ | ★ | ★ | 8 |
| Akinci et al, 2007 | ★ | ☆ | ★ | ★ | ★ | ★ | ☆ | ★ | ★ | ★ | 8 |
| Aksoy et al, 2021 | ★ | ☆ | ★ | ★ | ★ | ☆ | ☆ | ★ | ★ | ★ | 7 |
| Anderson et al, 1985 | ★ | ☆ | ★ | ★ | ★ | ☆ | ☆ | ★ | ★ | ★ | 7 |
| Ferdousi et al, 2018 | ★ | ☆ | ★ | ★ | ★ | ★ | ☆ | ★ | ★ | ★ | 8 |
| Goebbels et al, 2000 | ★ | ☆ | ★ | ★ | ★ | ★ | ☆ | ★ | ★ | ★ | 8 |
| Inanc et al, 2020 | ★ | ★ | ★ | ★ | ★ | ★ | ☆ | ★ | ★ | ★ | 9 |
| Koca et al, 2022 | ★ | ☆ | ★ | ★ | ★ | ★ | ☆ | ★ | ★ | ★ | 8 |
| Oriowo et al, 2009 | ★ | ☆ | ★ | ★ | ★ | ☆ | ☆ | ★ | ★ | ★ | 7 |
| Stolwijk et al, 1991 | ★ | ☆ | ★ | ★ | ★ | ☆ | ☆ | ★ | ★ | ★ | 7 |
| Symeonidis et al, 2013 | ★ | ☆ | ★ | ★ | ★ | ☆ | ☆ | ★ | ★ | ★ | 7 |
| Baek et al, 2015 | ★ | ☆ | ★ | ★ | ★ | ☆ | ☆ | ★ | ★ | ★ | 7 |
| Cakir et al, 2016 | ★ | ☆ | ★ | ★ | ★ | ☆ | ☆ | ★ | ★ | ★ | 7 |
| Celikay et al, 2021 | ★ | ☆ | ★ | ★ | ★ | ★ | ☆ | ★ | ★ | ★ | 8 |
| Chang et al, 1995 | ★ | ☆ | ★ | ★ | ★ | ☆ | ☆ | ★ | ★ | ★ | 7 |
| Cousen et al, 2007 | ★ | ☆ | ★ | ★ | ★ | ★ | ☆ | ★ | ★ | ★ | 8 |
| Dogru et al, 2004 | ★ | ☆ | ★ | ★ | ★ | ★ | ☆ | ★ | ★ | ★ | 8 |
| Dogru et al, 2001 | ★ | ☆ | ★ | ★ | ★ | ★ | ☆ | ★ | ★ | ★ | 8 |
| Fan et al, 2021 | ★ | ☆ | ★ | ★ | ★ | ☆ | ☆ | ★ | ★ | ★ | 7 |
| Gao et al, 2015 | ★ | ☆ | ★ | ★ | ★ | ☆ | ☆ | ★ | ★ | ★ | 7 |
| Han et al, 2021 | ★ | ★ | ★ | ★ | ★ | ☆ | ☆ | ★ | ★ | ★ | 8 |
| Kesarwani et al, 2017 | ★ | ☆ | ★ | ★ | ★ | ★ | ☆ | ★ | ★ | ★ | 7 |
| Li et al, 2014 | ★ | ☆ | ★ | ★ | ★ | ★ | ☆ | ★ | ★ | ★ | 8 |
| Liang et al, 2021 | ★ | ☆ | ★ | ★ | ★ | ☆ | ☆ | ★ | ★ | ★ | 7 |
| Lin et al, 2017 | ★ | ☆ | ★ | ★ | ★ | ★ | ☆ | ★ | ★ | ★ | 8 |
| Liu et al, 2019 | ★ | ☆ | ★ | ★ | ★ | ★ | ☆ | ★ | ★ | ★ | 8 |
| Liu et al, 2008 | ★ | ☆ | ★ | ★ | ★ | ★ | ☆ | ★ | ★ | ★ | 8 |
| Lyu et al, 2019 | ★ | ☆ | ★ | ★ | ★ | ★ | ☆ | ★ | ★ | ★ | 8 |
| Manchikanti et al, 2021 | ★ | ☆ | ★ | ★ | ★ | ★ | ☆ | ★ | ★ | ★ | 8 |
| Ozdemir et al, 2003 | ★ | ☆ | ★ | ★ | ★ | ★ | ☆ | ★ | ★ | ★ | 8 |
| Qu et al, 2017 | ★ | ☆ | ★ | ★ | ★ | ★ | ☆ | ★ | ★ | ★ | 8 |
| Saito et al, 2003 | ★ | ☆ | ★ | ★ | ★ | ☆ | ☆ | ★ | ★ | ★ | 7 |
| Sandra et al, 2019 | ★ | ☆ | ★ | ★ | ★ | ★ | ☆ | ★ | ★ | ★ | 8 |
| Stuard et al, 2017 | ★ | ☆ | ★ | ★ | ★ | ★ | ☆ | ★ | ★ | ★ | 8 |
| Toth et al, 2021 | ★ | ☆ | ★ | ★ | ★ | ☆ | ☆ | ★ | ★ | ★ | 7 |
| Trindade et al, 2021 | ★ | ☆ | ★ | ★ | ★ | ☆ | ☆ | ★ | ★ | ★ | 7 |
| Yang et al, 2018 | ★ | ☆ | ★ | ★ | ★ | ★ | ☆ | ★ | ★ | ★ | 8 |
| Yoon et al, 2004 | ★ | ☆ | ★ | ★ | ★ | ☆ | ☆ | ★ | ★ | ★ | 7 |
| Yu et al, 2008 | ★ | ☆ | ★ | ★ | ★ | ★ | ☆ | ★ | ★ | ★ | 8 |
| Yu et al, 2016 | ★ | ☆ | ★ | ★ | ★ | ★ | ☆ | ★ | ★ | ★ | 8 |
| Yusufu et al, 2018 | ★ | ☆ | ★ | ★ | ★ | ★ | ☆ | ★ | ★ | ★ | 8 |
| Zeng et al, 2019 | ★ | ☆ | ★ | ★ | ★ | ★ | ☆ | ★ | ★ | ★ | 8 |
| Zhang et al, 2020 | ★ | ☆ | ★ | ★ | ★ | ★ | ☆ | ★ | ★ | ★ | 8 |
| Zou X et al, 2020 | ★ | ☆ | ★ | ★ | ★ | ☆ | ☆ | ★ | ★ | ★ | 7 |
| Aljarousha et al, 2016 | ★ | ☆ | ★ | ★ | ★ | ★ | ☆ | ★ | ★ | ★ | 8 |
| Alves et al, 2014 | ★ | ☆ | ★ | ★ | ★ | ★ | ☆ | ★ | ★ | ★ | 8 |
| Beckman et al, 2014 | ★ | ☆ | ★ | ★ | ★ | ☆ | ☆ | ★ | ★ | ★ | 7 |
| De Freitas et al, 2021 | ★ | ☆ | ★ | ★ | ★ | ★ | ☆ | ★ | ★ | ★ | 8 |
| Derakhshan et al, 2019 | ★ | ☆ | ★ | ★ | ★ | ★ | ☆ | ★ | ★ | ★ | 8 |
| Eissa et al, 2016 | ★ | ☆ | ★ | ★ | ★ | ★ | ☆ | ★ | ★ | ★ | 8 |
| Garcia et al, 2018 | ★ | ☆ | ★ | ★ | ★ | ★ | ☆ | ★ | ★ | ★ | 8 |
| Gekka et al, 2004 | ★ | ☆ | ★ | ★ | ★ | ☆ | ☆ | ★ | ★ | ★ | 7 |
| Gunay et al, 2016 | ★ | ☆ | ★ | ★ | ★ | ★ | ☆ | ★ | ★ | ★ | 8 |
| Inoue et al, 2001 | ★ | ☆ | ★ | ★ | ★ | ☆ | ☆ | ★ | ★ | ★ | 7 |
| Onyekwelu et al, 2020 | ★ | ☆ | ★ | ★ | ★ | ★ | ☆ | ★ | ★ | ★ | 8 |
| Wang et al, 2021 | ★ | ☆ | ★ | ★ | ★ | ☆ | ☆ | ★ | ★ | ★ | 7 |
| Zhang et al, 2016 | ★ | ☆ | ★ | ★ | ★ | ★ | ☆ | ★ | ★ | ★ | 8 |
| Zhang et al, 2021 | ★ | ☆ | ★ | ★ | ★ | ★ | ☆ | ★ | ★ | ★ | 8 |
| Kan et al, 2018 | ★ | ☆ | ★ | ★ | ★ | ★ | ☆ | ★ | ★ | ★ | 8 |

**
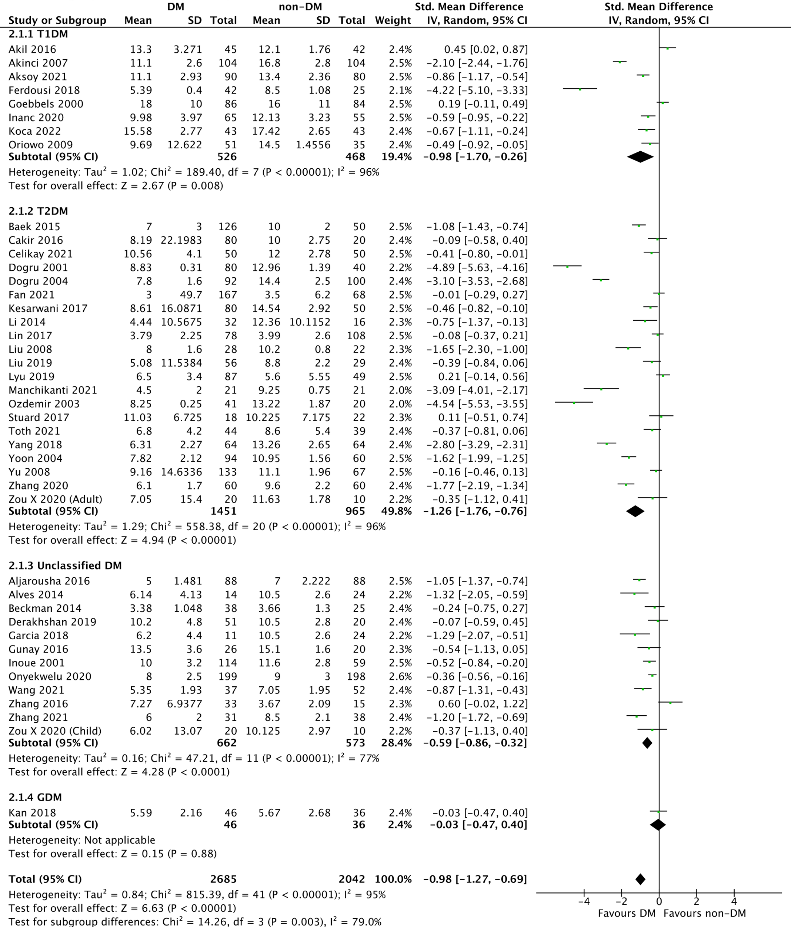
**

**Supplementary Figure 1A**

**
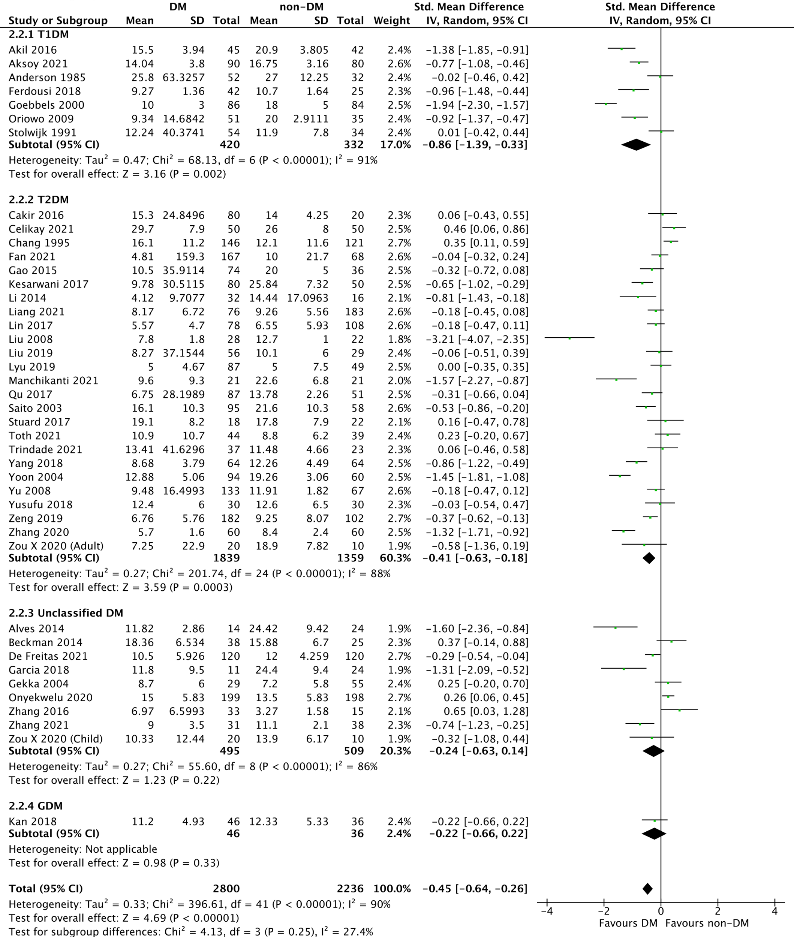
**

**Supplementary Figure 1B**

**Supplementary Figure 1.** Comparison of the severity of DED between different types of DM based on (A) ITBUT and (B) Schirmer’s 1 test.

**
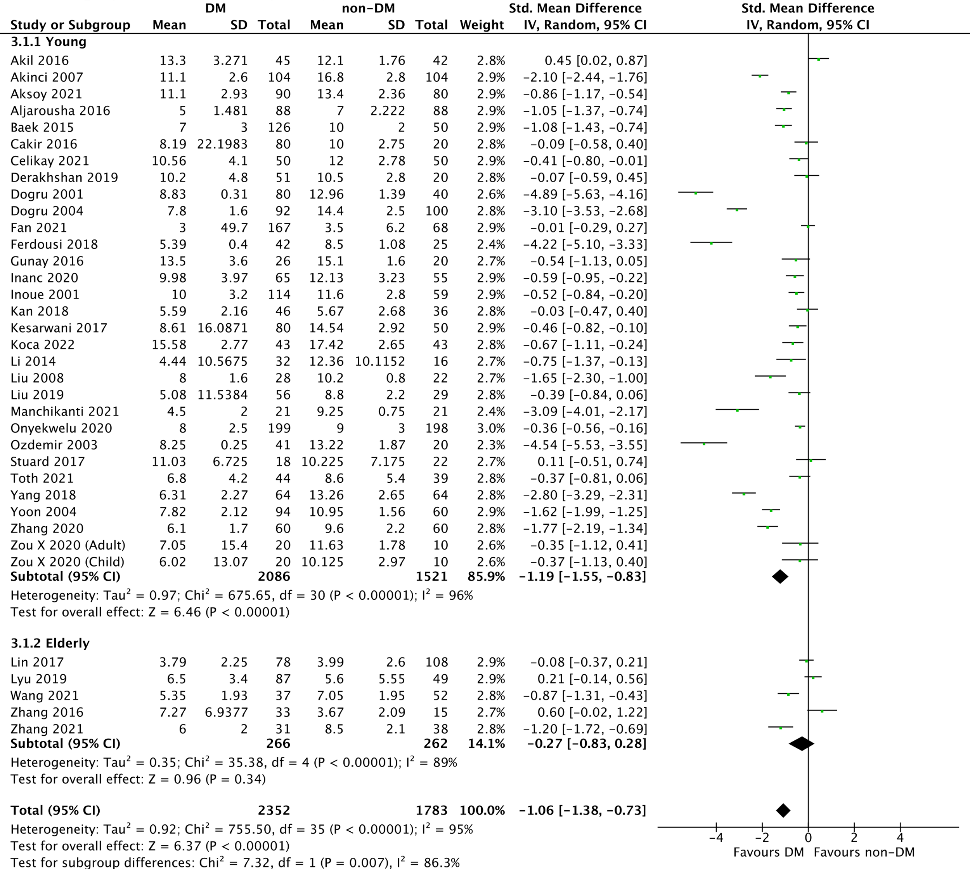
**

**Supplementary Figure 2A**

**
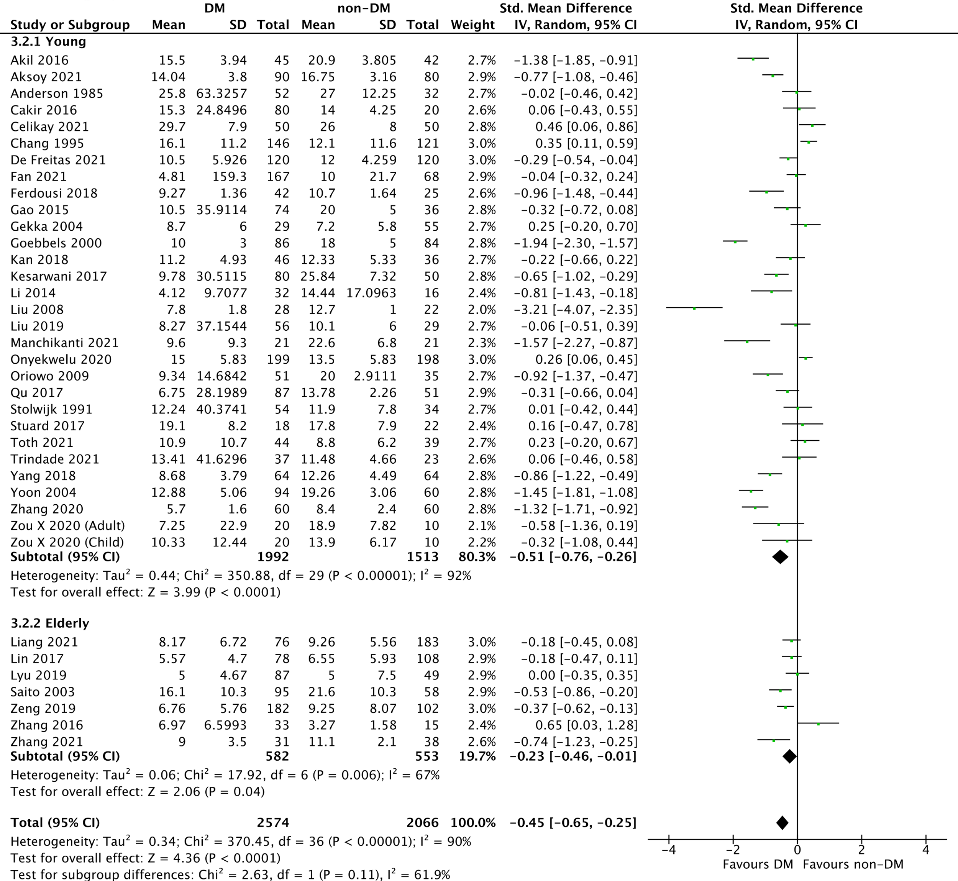
**

**Supplementary Figure 2B**

**Supplementary Figure 2.** Comparison of the severity of DED between different ages of DM based on (A) ITBUT and (B) Schirmer’s 1 test.

**
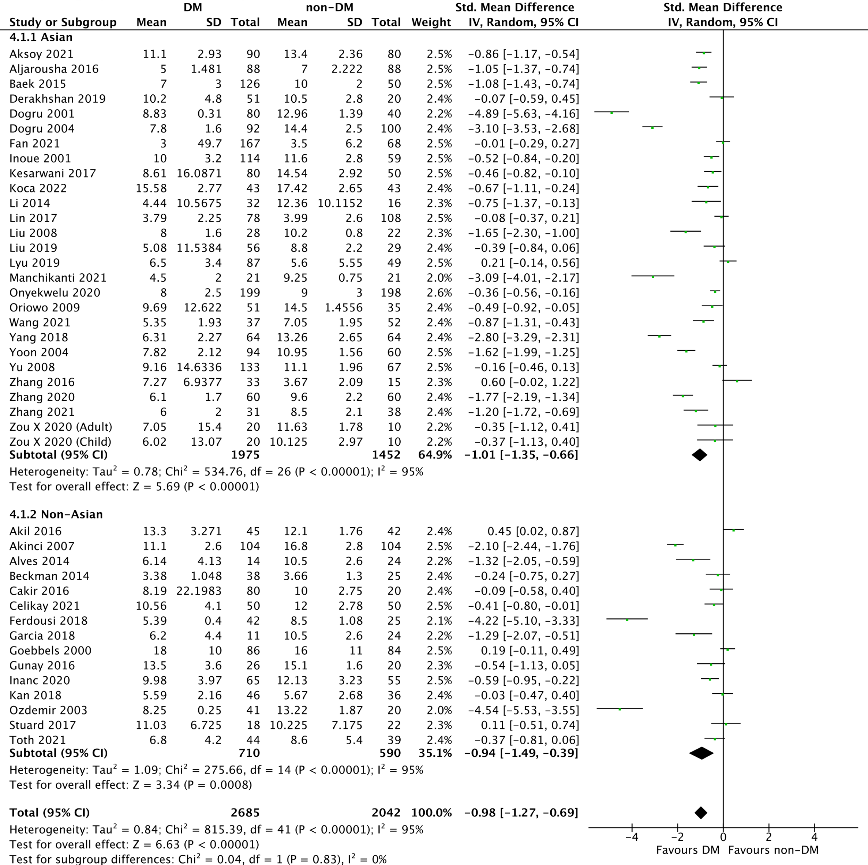
**

**Supplementary Figure 3A**

**
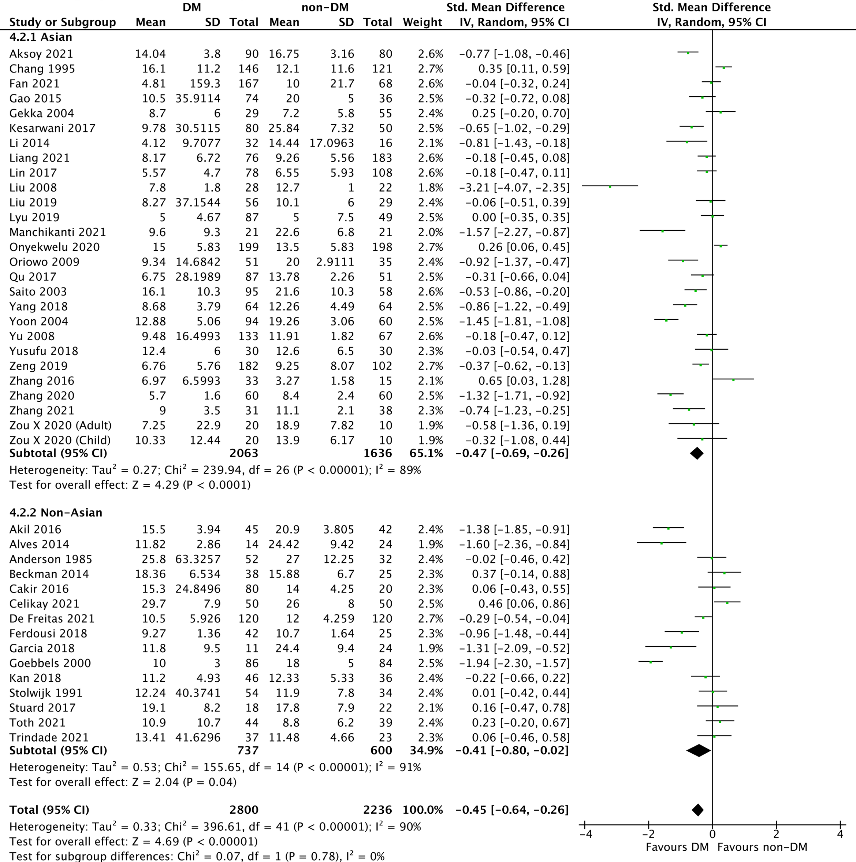
**

**Supplementary Figure 3B**

**Supplementary Figure 3.** Comparison of the severity of DED between different ethics of DM based on (A) ITBUT and (B) Schirmer’s 1 test.

**
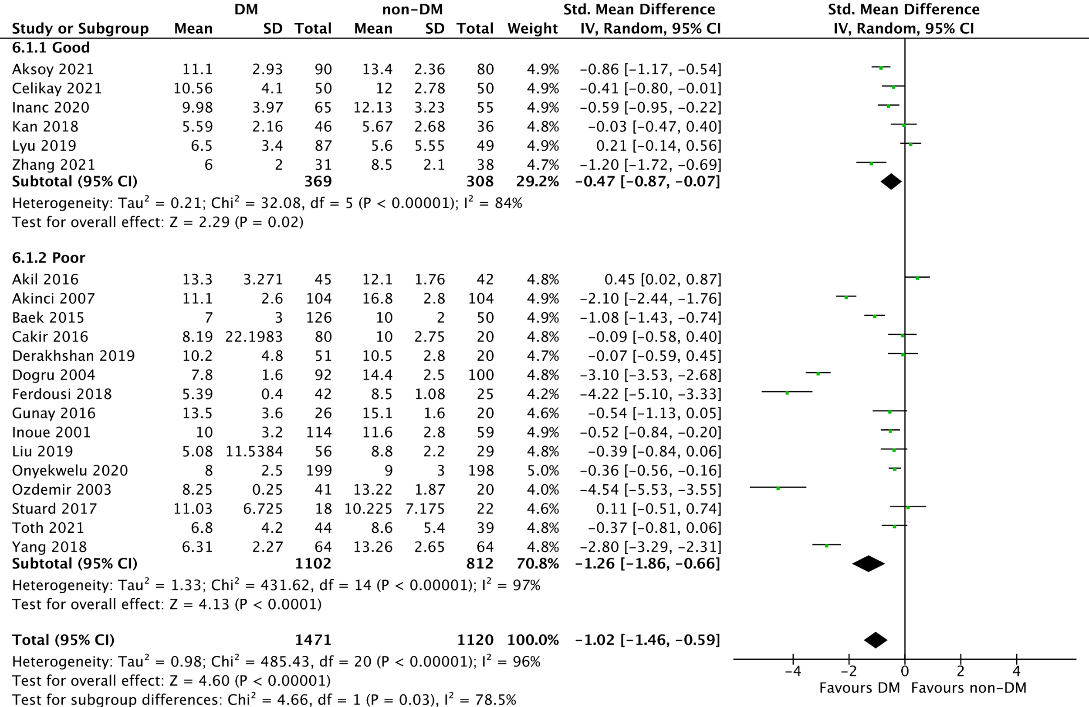
**

**Supplementary Figure 4A**

**
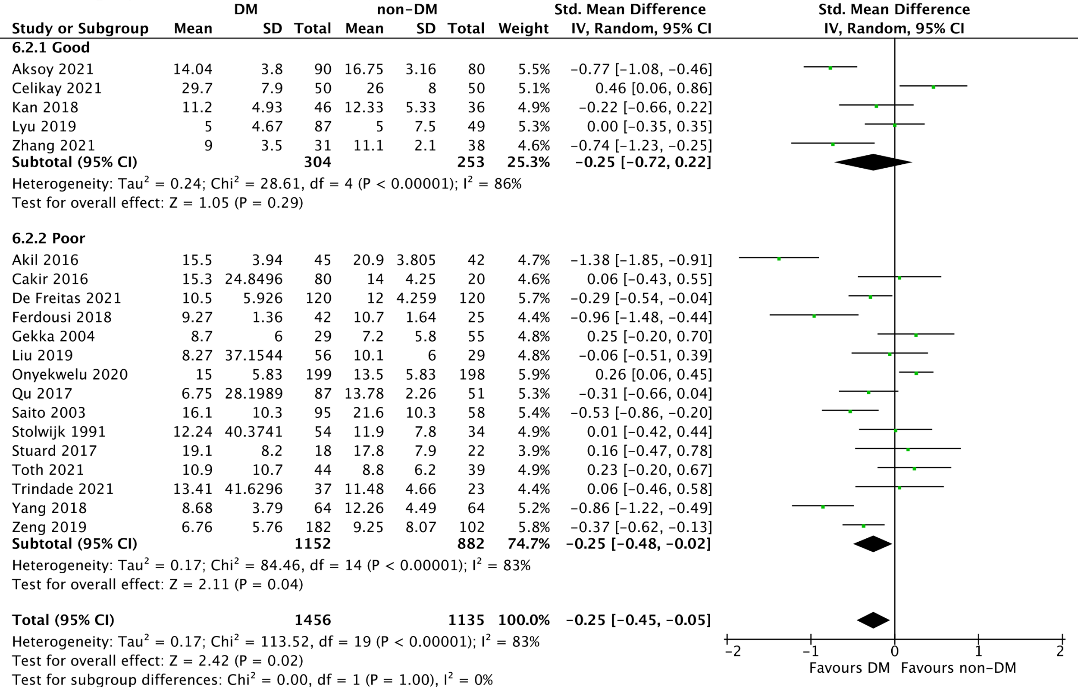
**

**Supplementary Figure 4B**

**Supplementary Figure 4.** Comparison of the severity of DED between different DM control status based on (A) ITBUT and (B) Schirmer’s 1 test.

**
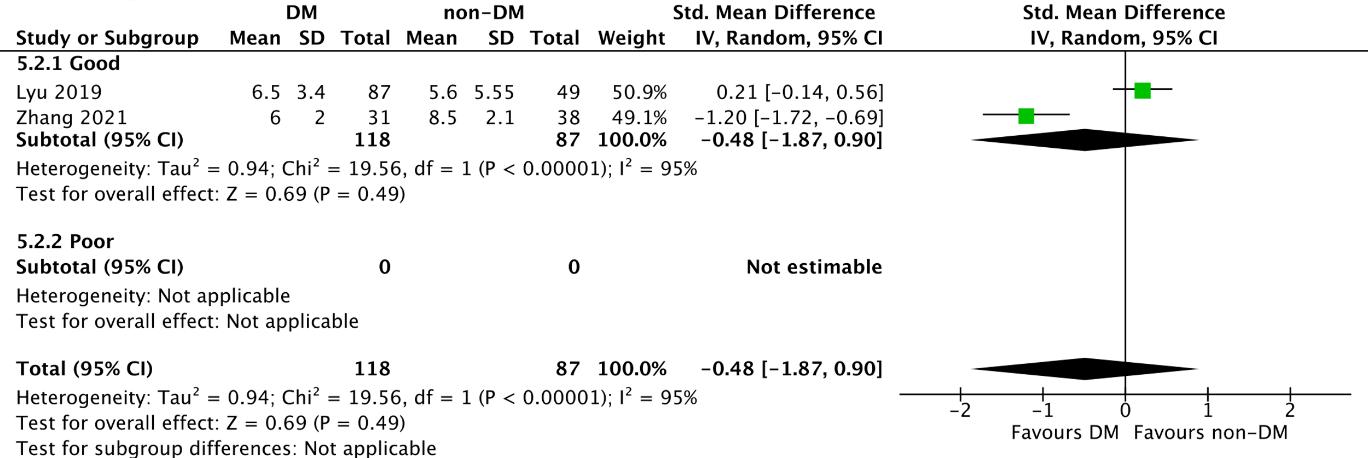
**

**Supplementary Figure 5A**

**
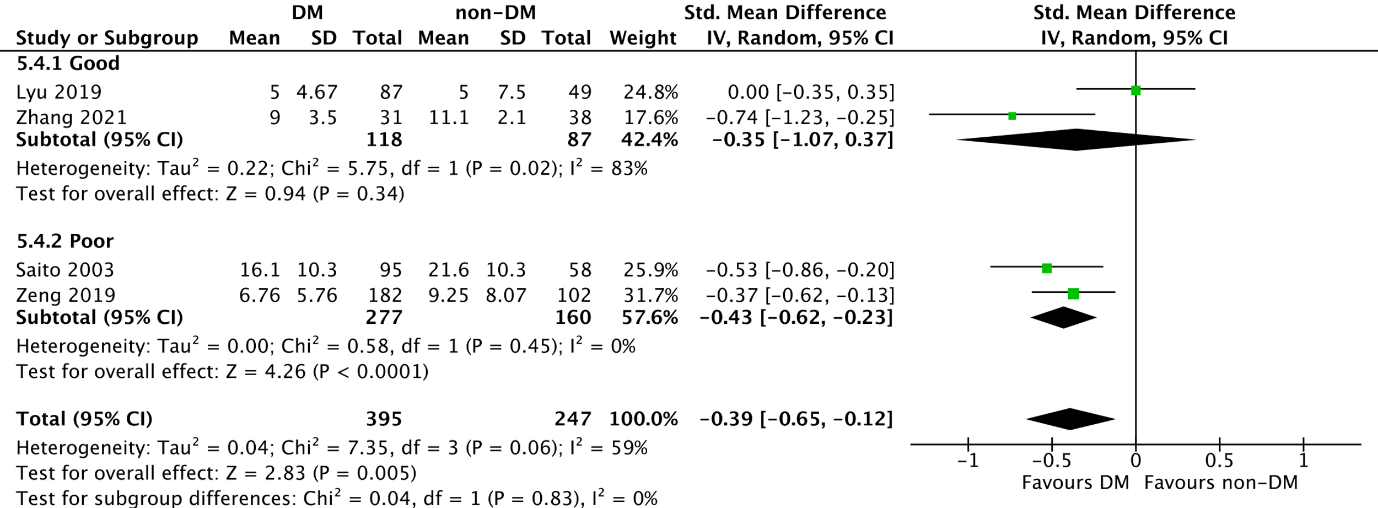
**

**Supplementary Figure 5B**

**Supplementary Figure 5.** Comparison of the severity of DED between different DM control status among elderly based on (A) ITBUT and (B) Schirmer’s 1 test.
